# Targeting sirtuins for cancer therapy: epigenetics modifications and beyond

**DOI:** 10.7150/thno.100667

**Published:** 2024-10-14

**Authors:** Hui Shen, Xinyi Qi, Yue Hu, Yi Wang, Jin Zhang, Zhongyu Liu, Zheng Qin

**Affiliations:** 1 Department of Respiratory and Critical Care Medicine, Department of Outpatient, The First Hospital of China Medical University, Shenyang 110001, China.; 2 Sichuan Engineering Research Center for Biomimetic Synthesis of Natural Drugs, School of Life Science and Engineering, Southwest Jiaotong University, Chengdu 610031, China.; 3 School of Pharmaceutical Sciences, Health Science Center, Shenzhen University, Shenzhen 518060, China.; 4 No. 989 Hospital of Joint Logistic Support Force of PLA, Luoyang 471031, China.

**Keywords:** Sirtuin (SIRT), Epigenetics modification, Cancer therapy, Small-molecule activator, Small-molecule inhibitor

## Abstract

Sirtuins (SIRTs) are well-known as nicotinic adenine dinucleotide^+^(NAD^+^)-dependent histone deacetylases, which are important epigenetic enzymes consisting of seven family members (SIRT1-7). Of note, SIRT1 and SIRT2 are distributed in the nucleus and cytoplasm, while SIRT3, SIRT4 and SIRT5 are localized in the mitochondria. SIRT6 and SIRT7 are distributed in the nucleus. SIRTs catalyze the deacetylation of various substrate proteins, thereby modulating numerous biological processes, including transcription, DNA repair and genome stability, metabolism, and signal transduction. Notably, accumulating evidence has recently underscored the multi-faceted roles of SIRTs in both the suppression and progression of various types of human cancers. Crucially, SIRTs have been emerging as promising therapeutic targets for cancer therapy. Thus, in this review, we not only present an overview of the molecular structure and function of SIRTs, but elucidate their intricate associations with oncogenesis. Additionally, we discuss the current landscape of small-molecule activators and inhibitors targeting SIRTs in the contexts of cancer and further elaborate their combination therapies, especially highlighting their prospective utility for future cancer drug development.

## Introduction

**Figure 1:**
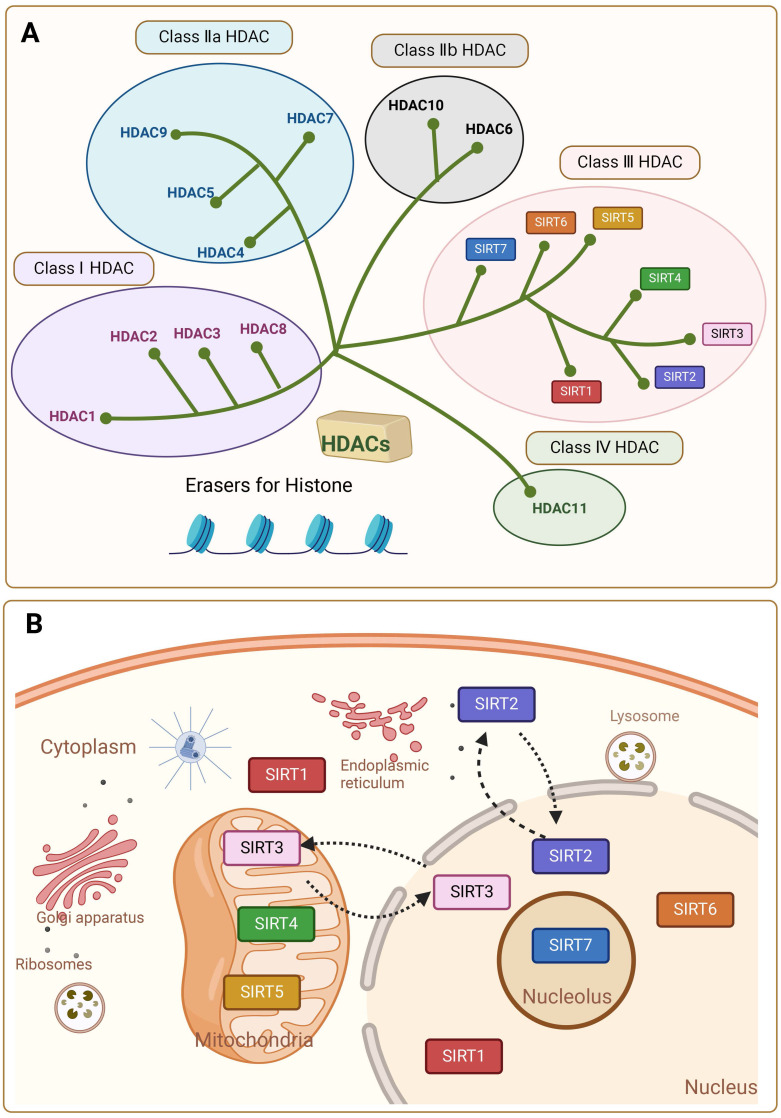
** The classification and subcellular localization of SIRTs.** (A) SIRTs are classified as the Class III HDAC. (B) SIRTs have seven members (SIRT1-7) and have different subcellular localization.

Sirtuins (SIRTs) are NAD^+^-dependent histone deacetylases (HDACs). SIRTs were first discovered and characterized in Saccharomyces cerevisiae and named the yeast silent information regulator 2 (Sir2), whose mammalian homologue is known as Sirt 1, 2. Other isoforms were later found in other species of bacteria, plants and mammals. HDACs are enzymes that eliminate acetyl groups from the lysine residues of histone and non-histone proteins. There are 18 known HDAC enzymes, which are divided into four major classes: Class I Rpd3-like proteins (HDAC1, HDAC2, HDAC3, and HDAC8); Class II Hda1-like proteins (HDAC4, HDAC5, HDAC6, HDAC7, HDAC9, and HDAC10); and Class III Sir2-like proteins (SIRT1, SIRT2, SIRT3, SIRT4, SIRT5, SIRT6, and SIRT7); and class IV proteins (HDAC11). In mammals the SIRTs family belongs to the evolutionary conserved proteins of class III histone deacetylases (HDAC) with seven members (SIRT1-SIRT7) **(Figure 1A)** 2. Around 275 amino acid residues make up the highly conserved central catalytic structural domain of SIRTs, which is encircled by structural domains at the C- and N-termini that differ in length and composition. Most NAD^+^/NADH-binding proteins have a Rossmann folded structure in the catalytic core structural domain. It also has a tiny globular structural domain with two insertion sites, one of which is for binding zinc ions and the other a helical module. In the deep cleft between the two structural domains of the enzyme is its active site 3. Variations in the distribution of SIRTs within organelles are caused by differences in the terminal level structures. In general, SIRT1 and SIRT2 are localized in the nucleus and cytoplasm, SIRT3, SIRT4 and SIRT5 in the mitochondria, and SIRT6 and SIRT7 only in the nucleus 4, 5. SIRT1 is predominantly found in the nucleus but can be transported to the cytoplasm. SIRT2 can shuttle to the nucleus, but is mostly detected in the cytoplasmic lysate 5** (Figure 1B)**. This affects their diversity in terms of substrate specificity, binding to intracellular regulatory molecules, and enzymatic activity of various SIRTs.

Histone acetylation is an important epigenetic mechanism that plays an important role in the cell cycle and cell differentiation by turning chromatin on and off to alter chromatin structure and regulate gene expression 6. Histone acetylation is regulated by two types of enzymes: histone acetyltransferases (HAT) and HDACs. Histone deacetylase catalyzes the removal of acetyl groups from ε-N-acetyllysine residues of histones and non-histone proteins, thereby balancing the action of HAT 7. Deacetylation affects a multitude of significant biological processes, including DNA recombination and repair, transcriptional silencing, lipid mobilization, apoptosis, and senescence. SIRTs are class III histone deacetylases that require NAD^+^ as a co-substrate for catalytic activity 8. Deacetylation by SIRTs induces epigenetic changes and regulates transcription and DNA repair. Additionally, SIRTs have been reported to function as demyristoylases, lipoamidases and desuccinylases, demalonylases and deglutarylases 9.

Consequently, SIRTs have been implicated in numerous vital biological processes, encompassing cellular proliferation and differentiation, cell adhesion, intercellular communication, gene transcriptional regulation, energy metabolism modulation, stress response orchestration, inflammation control, cancer progression facilitation and metastasis promotion 8. The latest evidence indicates that aberrant expression of SIRTs is observed in almost all cancer types with distinct mechanisms implicated in cancer metabolism, genome stability, and the tumor microenvironment. The remarkable aspect is that SIRTs exhibit a dual function in cancer, acting as cancer suppressor and promoter under different conditions. In addition, SIRTs can play a regulatory role in tumor response to chemotherapy. The distinctive characteristics imply that SIRTs have the potential to serve as targeting markers and play an important role in cancer therapy 10. At present, inhibitors and activators have been developed for SIRTs, but are mainly preclinical small molecule modulators. In general, activators exhibit superior therapeutic potential compared to inhibitors partly due to their higher target specificity and reduced side effects within the enzyme family. However, the availability of activators is limited in comparison to inhibitors. Therefore, the development of modulators with clinical applications and activator classes holds significant potential.

In this review, we aim to elucidate the structure and functionality of SIRTs, with a particular emphasis on providing a comprehensive overview of the biochemical processes associated with the cellular functions of the SIRT family. In addition, we elucidate the epigenetic and beyond functions of SIRTs in cancer, and provide a comprehensive overview of the small molecule drugs that target SIRTs in cancer therapy. Additionally, considering the current preclinical research status of SIRTs-targeted compounds, we address the limitations in the existing SIRT family with respect to tumor pathogenesis research and candidate drug development. Furthermore, we highlight the significant potential of targeting SIRTs in cancer therapy.

## The structure, function and regulation of SIRTs

### The structure and function of SIRTs

**Figure 2:**
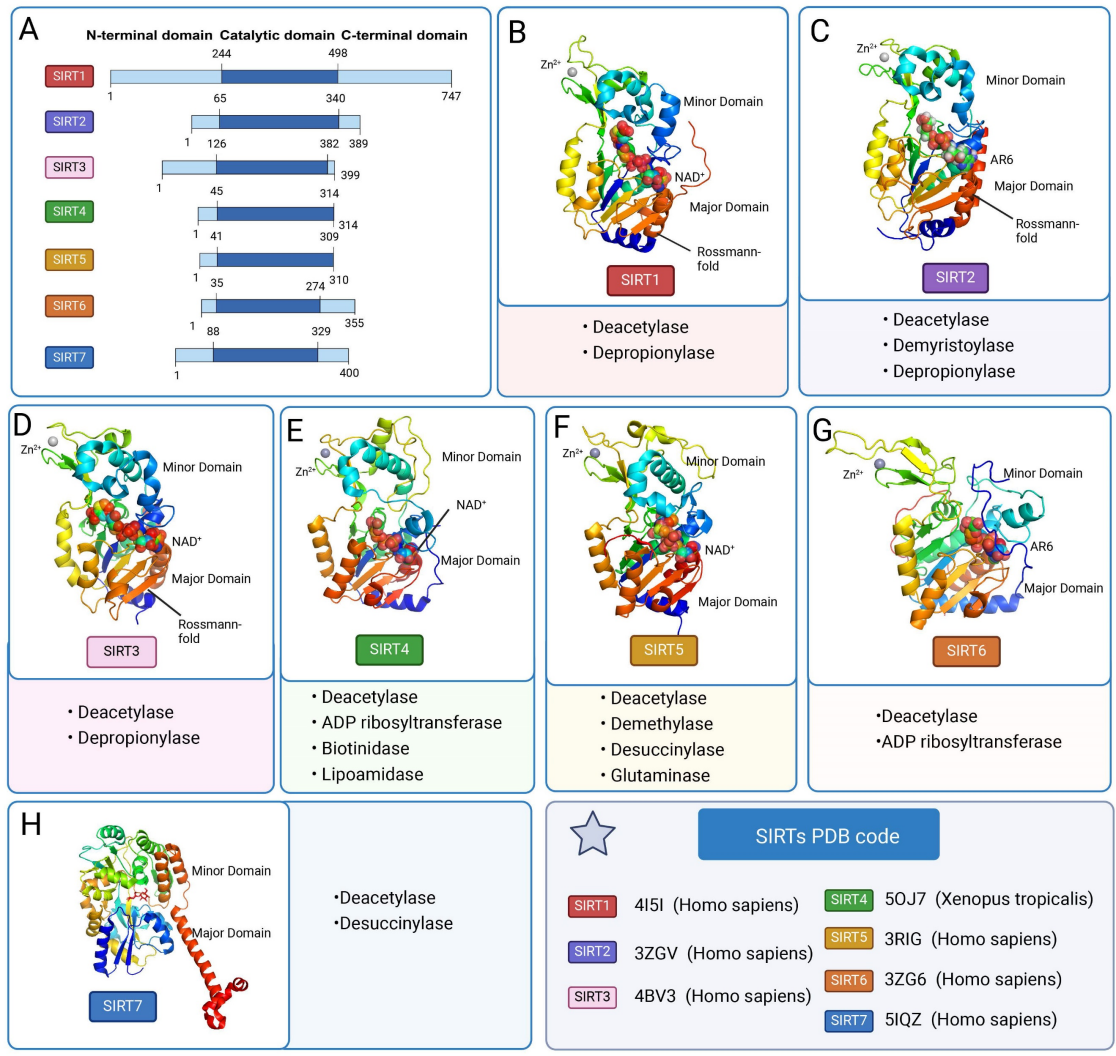
** The classification and structure of SIRTs.** (A) Domain organization of SIRTs. (B-H) The three-dimensional structure of SIRTs and basic function. (SIRT1 PDB: 4I5I; SIRT2: 3ZGV; SIRT3: 4BV3; SIRT4: 5OJ7; SIRT5: 3RIG; SIRT6: 3ZG6; SIRT7: 5IQZ).

SIRTs contain a conserved catalytic core area as well as varied C-terminal and C-terminal regions. The two primary components of the catalytic core area are the large structurally homologous Rossmann folding domain and the small structurally more varied zinc ion domain 11. A big groove is formed by three large structural domain polypeptide chains and a tiny structural domain polypeptide chain connecting these two structural domains 12. The Rossmann-fold structural domain is a typical NAD^+^ binding site. The structure is made up of six parallel β-strands that form a core β-sheet, as well as several α-helices on both sides. The zinc ion-binding structural domain identifies the particular architectures for various SIRTs, which are determined by their primary sequence, three-dimensional structure, and position relative to the larger structural domain. The minor structural domain is composed of two modules: a SIRT-dependent variable α-helix region and a three-stranded antiparallel β-sheet. The cofactor-binding loop area is the cleft that connects the four loops of the small and large structural domains to form the enzyme active site 13. NAD^+^ and peptide substrates with acetyl lysine enter and bind to the enzyme. Beneath the two structural domains is a protein channel that contains the reactive groups of the two bound substrate molecules and the amino acids responsible for catalysis 14. Because of the high sequence homology in this region of the protein family, mutations at these locations will directly cause the catalytic activity of deacetylation to be lost. In the enzymatic conformation of SIRTs, the largest of four loops (β1-α2 loop in Hst2) usually serves as one of the most active areas 11. In addition to the conserved catalytic core region, the variable N-terminal and C-terminal regions vary in length, sequence, and secondary structure, and are not conserved within the protein family **(Figure 2 A)**.

Although the SIRTs family has a similar protein folding structure, it has different specific conformations. SIRT1 differs from other family members in having large disordered C-terminal domains. The catalytic core and metastable site are situated between residues 181 and 498, forming a compact globular core. The structural domain of NAD^+^ binding consists of eight α-helices (αA, αB, αG, αH, and αJ-αM) stacked on the β-sheet core, and a central six-stranded parallel β-sheet (β1-β3 and β7-β9 chains). The small structural domain of the zinc ion consists of three β-strands, β4 to β6 and one helix, four invariant cysteine residues Cys371, Cys374, Cys395, and Cys398 tetrahedrally coordinated. The helical module consists of four α helices (αC-F). The CTR complements the β sheet of the NAD^+^ binding domain by forming a β hairpin shape over an essentially unchanging hydrophobic area 15. SIRT2 has a catalytic core of 304 amino acids and a 19-residue N-terminal helical extension. The NAD-bound macrostructural domain consists of six β-strands (β1-3 and β7-9) and six α-helices (α1, α7, α8, and α10-α12). The former form parallel β-sheets, while the latter are apposed to the β-sheets. The small structural domain of the zinc ion, consisting of four α-helices (α 3-α6), three strands of antiparallel β-folds (β4, β5, β6), α-helices (α9) and zinc atoms tetrahedrally coordinated by four Cys residues (C195, C200, C221, C224). SIRT3 is structurally similar to other family members but has a C-terminal structural domain that targets mitochondria. The crystal structure of human SIRT4 has not been studied yet. The SIRT5 macrostructural domain consists of six parallel β strands (β1-β3 and β7-β9) and a core β sheet of nine α helices (α1, α2, α6, α7 and α10-α14). The minor structural domain consists of five α helices (α3-α5, α8 and α9) and a three-stranded antiparallel β sheet (β4, β5 and β6). Four aminocysteine residues (C166, C169, C207, and C212) coordinate the zinc ion in a tetrahedral configuration. Nestled between the α2 helix of the zinc binding domain and the α3 helix of the Rossmann folding domain lies the N ring, which is intimately associated with NAD^+^ binding. Substrate binding is carried out by the S ring, which is situated between the α10 helix of the Rossmann folding domain and the β6 chain of the zinc-binding domain 16. The large structural domain of SIRT6 consists of a six-stranded parallel β-sheet (β1, β2, β3, β7, β8, β9) located between two helices on one side (α6, α7) and four helices on the other side (α1, α4, α5, α8). The minor structural domain consists of two extension loops (β3, α6) and a three-stranded antiparallel β-sheet (β4, β5, β6). SIRT6 has both N- and C-terminal extensions. Compared to other SIRTs, SIRT7 and SIRT6 lack a bigger helix in the NAD^+^-binding Rossmann folding region. This modification drastically lowers the flexibility of the structure and consequently the deacetylase activity 17.

The SIRTs specifically target a diverse range of cellular proteins and transcription factors within the nucleus, cytoplasm, and mitochondria, thereby playing a crucial role in the regulation of epigenetic processes 18. The deacetylation mediated by SIRTs is considered one of the most prominent epigenetic mechanisms. The function of SIRTs in nature is opposite to that of an acetyltransferase, as it involves the removal of acetyl modifications from lysine residues 19. Protein acetylation is an essential post-translational modification that modifies the surface charge and structure of proteins, hence affecting their function 12. Deacetylation by SIRTs is an important epigenetic alteration. The catalytic activity of SIRTs protein family members is controlled by the following processes: first, NAD^+^ and acetyl lysine substrate binding; second, glycosidic bond cleavage and acetyl transfer; and finally, the synthesis of O-acetyl-ADPR, nicotinamide, and deacetylated lysine products 10. SIRTs consume one NAD^+^ molecule to convert it to 2′-O-acetyl nicotinamide and ADP-ribose in each lysine deacetylation reaction 20. Among them, SIRT1-3 have potent deacetylating activity, and SIRT4-7 are less active. SIRT3 is the primary mitochondrial deacetylase among the three mitochondrial SIRTs 21. Unlike other SIRTs, SIRT6 exerts its deacetylation according to packed nucleosomes rather than free histones.

The initial designation of SIRT as a deacetylase was subsequently expanded to encompass its capability for additional modifications beyond deacetylation 22. SIRT1, SIRT2 and SIRT3 exhibit depropionylase activity 23. SIRT2 has demyristoylation activity. Its activity for eliminating long-chain fatty acyl groups is effective, and its catalytic efficiency for eliminating myristoyl groups is somewhat greater than that of eliminating acetyl groups 24. In mitochondria, SIRT4 is involved in lipoamylase, biotinidase, and ADP ribosyltransferase activities 25. Together with NAD^+^-dependent protein lysine demethylase, desuccinylase, and lysine glutaminase activities, SIRT5 also exhibits modest deacetylase activity 26. SIRT5 inhibition increases glutaminase succinylation, which is the enzyme responsible for converting glutamine to glutamate. Moreover, SIRT6 has *in vitro* deacetylase activity and NAD^+^-dependent mono-ADP ribosyltransferase activity 27. SIRT7 is a comparatively little-studied enzyme that was first discovered to have very little enzymatic activity as a deacetylase 28. SIRT7 was later identified as activated by DNA in the chromatin environment to regulate ribosomal DNA transcription, RNA polymerase I, and chromatin remodeling 29. In addition, SIRT7 was discovered to be a NAD^+^-dependent histone desuccinylase. SIRT7 desuccinylates H3K122 to promote chromatin condensation and DNA repair 30. In brief, SIRTs actively participate in a diverse range of biological processes, including metabolic regulation, chromatin remodeling, RNA transcription, inflammation, cell cycle control, cellular differentiation, programmed cell death, immune response, oxidative stress management, DNA repair, cellular respiration regulation and optimization, as well as microtubule dynamics modulation 31** (Figure 2, Table 1)**.

### The regulation of SIRTs

**Figure 3:**
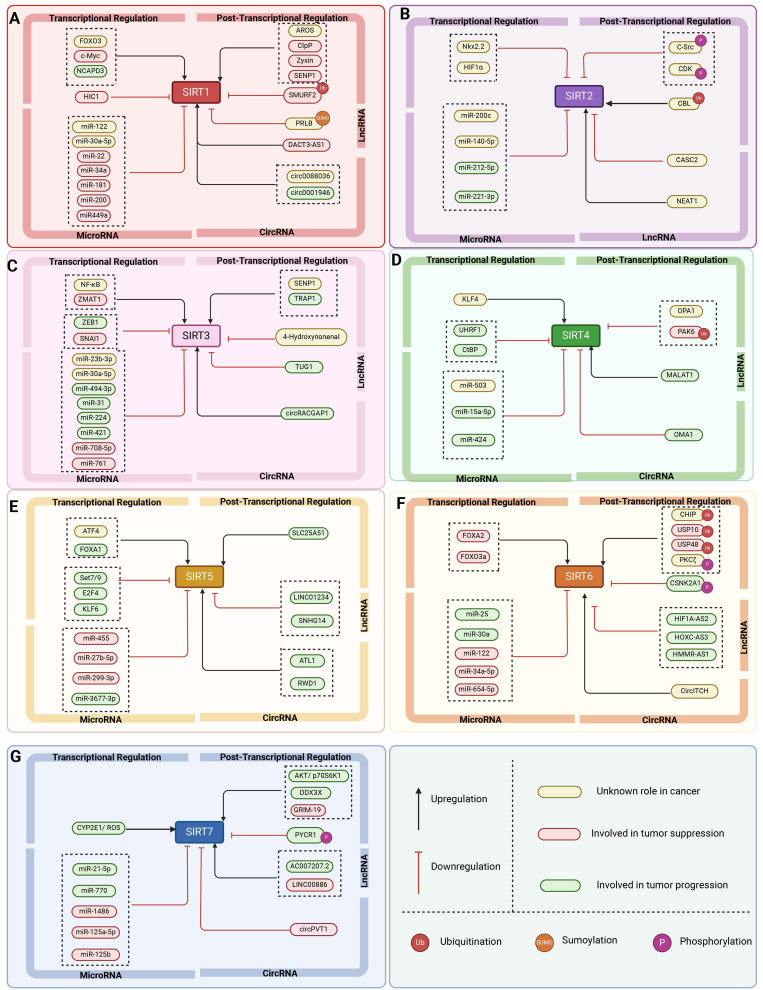
** The regulation of SIRTs in cancer.** The transcriptional regulation, post-transcriptional regulation, RNA-mediated regulation, protein regulation and other regulation of SIRT1-7.

The expression and activity of SIRTs are regulated at multiple levels, encompassing transcriptional control, translational regulation, post-translational modifications, impact on protein stability, and additional regulatory mechanisms **(Figure 3, Table 2)**. Multiple transcription factors have an effect the expression level of SIRTs. For example, during acute nutritional stress, the transcription factor Forkhead box protein O3 (FOXO3a) enhances the transcriptional expression of SIRT1 by interacting with p53 at the p53 binding site in the SIRT1 promoter 39. Furthermore, NCAPD3 accelerates the progression of diffuse large B-cell lymphoma by recognizing H3K9 monomethylation (H3K9me1) on the SIRT1 promoter, which is connected to the SIRT1 promoter by TFII I, and increasing SIRT1 transcription 40. Hypoxia-inducible factor 1α (HIF1α) can repress SIRT2 gene transcription by interacting with the evolutionarily conserved hypoxia response element (HRE) in the SIRT2 promoter 41.

SIRTs are also regulated by transcriptional modifications. Of which microRNAs (miRNAs) are post-transcriptional regulators of mRNA stability and protein levels that modulate the activity of SIRTs. For example, the tumor suppressor (HUR) binds to the mRNA of SIRT1 and improves its stability. miR-22, miR-34a, and miR-200a can reduce SIRT1 expression by preventing mRNA translation or encouraging mRNA degradation 39. Other miRNAs, including miR-449a, have been examined in models of acute kidney damage by measuring the expression of their target, SIRT1, and downstream proteins, p53/Bax. Inhibiting miR-449 enhances SIRT1 expression and decreases acetylated p53 and Bax protein levels 42. MiR-200c-5p targets SIRT2 and suppresses its expression. The MiR-200c-SIRT2 axis plays a critical role in metabolic reprogramming (Warburg-like effect) during human induced pluripotency and pluripotent stem cell activity via regulating glycolytic enzymes 43. MiR-15a-5p exacerbates the malignant phenotype of colorectal cancer cells by inhibiting SIRT4 expression through regulating STAT3/TWIST1 and PETN/AKT signaling in CRC cells 44. Furthermore, circLRWD1 regulates SIRT5 expression by adsorbing miR-507. That is, SIRT5 overexpression limits circLRWD1 silencing-mediated reduction of DDP resistance in DDP-resistant non-small cell lung cancer (NSCLC) cells 45.

Transcribed but untranslated long-stranded noncoding RNA (lncRNA) genes, more than 200 nucleotides in length, are categorized as a separate class of nonprotein-coding genes that can encode small functionalities that can modulate the activity of SIRTs. For instance, LINC01234 targets SIRT5 and regulates ovarian cancer (OS) development through miR-27b-5p. LINC01234 down-regulates or elevates miR-27b-5p to inhibit the progression of OCSCs and tumorigenesis *in vivo*. In addition, LINC01234 can restore SIRT5 expression by binding to miR-27b-5p 46. HIF1A-AS2 interacts with miR-33b-5p, inhibiting its expression and boosting the protein expression of the miR-33b-5p target SIRT6. *In vivo* investigations have demonstrated that downregulating HIF1A-AS2 decreased tumor growth in OS 47.

In addition, circular RNAs (circRNAs) are a novel group of endogenous non-coding RNAs that regulate gene expression in eukaryotes. CircRNAs regulate SIRTs by acting as miRNA sponges, which can be linked to cancer development. For example, SIRT1 overexpression reversed the inhibitory effect of circ_0001946 knockdown on the Wnt/β-catenin signaling pathway and promoted lung adenocarcinoma progression 48. CircOMA1 may act as an oncogenic circRNA in BC by regulating the miR-1276/SIRT4 axis 49. Another study discovered that the tumor suppressor human cyclic RNA CircITCH sponge miR-330-5p decreased adriamycin-induced cardiotoxicity by elevating SIRT6, survivin, and SERCA2a 50.

It's worth noting that SIRTs are also regulated by post-translational modification (PTM) such as phosphorylation, acetylation, sumoylation, methylation, ubiquitination, carbonylation, S-nitrosylation, S-glutathionylation, S-sulphenylation and nitration. Importantly, PTM can influence Sirtuin protein subcellular distribution and activation, as well as proteasomal degradation. GSK3β, a serine/threonine protein kinase, phosphorylates SIRT2 at three specific serine residues (S327, S331, and S335), which improves SIRT2 deacetylase activity. On the other hand, carbonylation of SIRT3 at the critical cysteine residue at position 280 (C280) is considered to be an inhibitory post-translational modification, as this modifying variant inhibits SIRT3 deacetylase activity 51. SRC proto-oncogene, nonreceptor tyrosine kinase (C-Src), phosphorylates SIRT2 at Tyr104 and affects its stability. CDK phosphorylates SIRT2 at the Ser331 location, which reduces its catalytic activity. Furthermore, Casitas B-lineage lymphoma (CBL) enhances the protein amount and stability of SIRT2 via ubiquitination 41.

Protein-protein networks are also another major method for regulating SIRTs. The mitochondrial protease ClpP activation regulates vascular smooth muscle cells by modifying the cellular NAD^+^/NADH ratio and activating SIRT1 to regulate aneurysm development 52. C-terminal binding protein (CtBP) promotes glutamine catabolism by directly suppressing the production of SIRT4, a glutamine catabolism repressor that enzymatically modifies glutamate dehydrogenase in mitochondria, in cancer cells. Loss of CtBP in cancer cells increases apoptosis due to intracellular acidification, as well as the disturbance of metabolic balance in cancer cells, as demonstrated by glutamine depletion, oxidative phosphorylation, and reduced ATP generation 53. Moreover, solute carrier family 25 member 20 (SLC25A51) is a recently identified mammalian mitochondrial NAD transporter. Overexpression of SLC25A51 promotes the advancement of hepatocellular carcinoma (HCC) by boosting aerobic glycolysis via SIRT5 54. Furthermore, SIRT7 interacts with kinesin family member 23 (KIF23) to improve KIF23 protein stability and stimulate cell motility by succinylating the K537 site of KIF23 55.

## The multi-faceted roles of SIRTs in cancer: epigenetic modifications and others

The SIRTs are notably involved in chromatin regulation, cellular survival, metabolic homeostasis, cell development and differentiation, as well as aging. Their involvement in the regulation of gene transcription, metabolism, adipose tissue mobilization, DNA damage repair, apoptosis, and other vital biological processes is crucial for tumor growth. With the deepening of research, it is increasingly recognized that SIRTs have a dual role in cancer, function as both oncogenes and tumor suppressors. This dual role reflects the complex relationship between SIRTs and cancer and emphasizes the potential of targeting SIRTs for cancer treatment (**Table 3 and Table 4**).

### Tumor-suppressing

#### SIRT1

SIRT1 inhibits cancer progression mainly by affecting DNA repair and increasing genomic stability primarily through substrate deacetylation. Activation of SIRT1 deacetylates Purine/pyrimidine-free nucleic acid endonuclease-1 (APE1) and promotes the binding of APE1 to the BER protein X-ray cross-complementarity-1 (XRCC1), thereby regulating cellular base excision repair. Knockdown of *SIRT1* increases the amount of cellular base-less DNA and sensitizes cells to genotoxic stress-induced death, a vulnerability that can be rescued by *APE1* overexpression 116. Furthermore, a key component of oncogenicity is XPA, a core nucleotide excision repair (NER) element required for the NER process. SIRT1 deacetylates XPA at lysine 63 and 67 sites, thereby enhancing NER activity 117. Furthermore, SIRT1 deacetylates and thus inhibits XPC transcription, and SIRT1 impairs global genomic NER. SIRT1 increases XPC expression through decreasing the AKT-dependent nuclear localization of XPC transcriptional repressor proteins 118. Additionally, SIRT1 interacts with the PP4 phosphatase complex and facilitates its inhibition by deacetylating the WH1 structural domain of the regulatory subunit PP4R3α/β, thereby regulating DNA repair and maintaining genome stability 119. SIRT1 is also associated with promoting double-strand break (DSB) repair and single-stranded DNA damage repair. In chronic myeloid leukemia (CML) cell lines, binding of SIRT1 to DNA damage sites induced by DSBs promotes homologous recombination (HR) through the Werner helicase (WRN) 120. SIRT1 deacetylates Sterile alpha motif and HD domain-containing protein 1 (SAMHD1) at K354, which binds directly to the ssDNA of DNA DSB and promotes DNA terminal excision and HR, which in turn promotes CtIP ssDNA binding to promote genome integrity 121. In addition to HR, SIRT1 promotes nonhomologous end joining (NHEJ) in CML cells through deacetylating Ku70 122.

**Figure 4:**
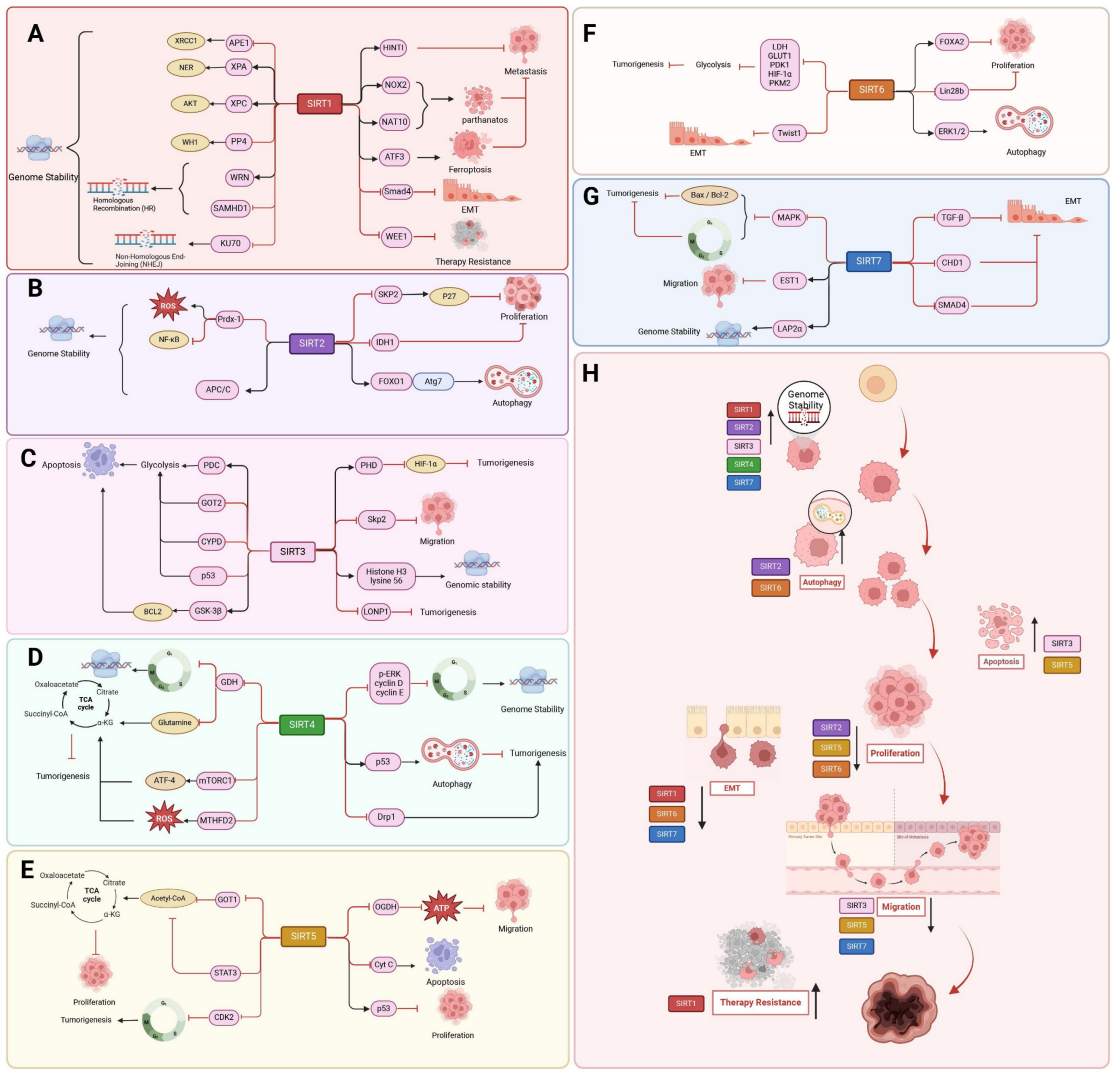
** The tumor suppressor role of SIRT1-7.** SIRT1-7 can regulate different tumor processes by regulating different substrates, affecting tumor genomic stability, proliferation, metastasis, metabolism, response to tumor therapy and others.

SIRT1 also regulates cellular proliferation and migration, which has an inhibitory influence on the progression of cancer. In colon cancer and melanoma cells, deacetylation by SIRT1 promotes tumor suppressor activity by enhancing the ability of histidine triad nucleotide-binding protein 1 (HINT1) to bind to β-catenin or MITF, resulting in an increase in the tumor suppressor function of HINT1^123^. In addition, programmed cell death induction is a novel finding in the use of SIRT1 to inhibit tumor progression. Activated SIRT1 promotes deoxypodophyllotoxin (DPT)-induced parthanatos in human glioma cells by initiating NAD^+^ depletion-dependent upregulation of NADPH oxidase 2 (NOX2) and N-acetyltransferase 10 (NAT10) 124. Additionally, SIRT1 plays a role in the occurrence of ferroptosis in cancer. By depleting NAD^+^ to facilitate ATF3 activation and inhibiting SLC7A11 and GPX4, SIRT1 can enhance the susceptibility of glioma cells to ferroptosis 125. SIRT1 inhibits EMT in HMLER breast cancer cells by deacetylating Smad4 and preventing TGF-β signaling 126. Interestingly, SIRT1 also deacetylates the cell-cycle checkpoint kinase WEE1, sensitizing cancer cells to WEE1 inhibition **(Figure 4 A, H)** 127.

#### SIRT2

As a tumor suppressor, SIRT2 prevents the growth of tumor cells in a variety of malignancies, such as colorectal, breast, prostate, human glioma, and cervical squamous cell carcinoma 128. Through deacetylation, SIRT2 can prevent the growth of cancer. SIRT2 overexpression in NSCLC enhanced the expression level of p27, a cell cycle protein dependent kinase inhibitor, and hindered the proliferation of NSCLC cells by promoting the deacetylation and degradation of S-phase kinase-associated protein 2 (Skp2). Histone deacetylase and proteasome inhibitors significantly inhibited the deacetylation of Skp2 by SIRT2 and the degradation of p27 by Skp2 129. The peroxidase activity of antioxidants is reduced by SIRT2, leading to the deacetylation of the antioxidant protein peroxiredoxin (Prdx-1). Consequently, breast cancer cells overexpressing SIRT2 accumulate ROS and experience DNA damage, resulting in decreased viability, particularly under H_2_O_2_-induced oxidative stress 130. Furthermore, Prdx-1 inhibits the transcription of TNFα-mediated NF-κB 131. The role of SIRT2 in maintaining mitosis and genome integrity involves the regulation of APC/C activity through deacetylation of co-activating proteins CDH1 and CDC20, thereby modulating their interaction with CDC27. In contrast, deletion of *SIRT2* leads to elevated levels of many mitotic regulators and may contribute to spontaneous tumor formation, with females developing primarily breast cancer and males developing primarily HCC 132. Increased expression of SIRT2 considerably reduces the proliferation, migration, and invasion of colorectal cancer cells by controlling the acetylation of isocitrate dehydrogenase 1 (IDH1) at the K224. Furthermore, IDH1 acetylation inhibited HIF1α-dependent SRC transcription, which managed the advancement of CRC 133. Interesting, AKT activation via the mTORC2 complex is induced by phosphokinase activity that is guaranteed by acetylation of non-histone MAPK/P38, a new substrate for SIRT2. In neuroblastoma cells, Sirt2-SUMOylation controls the level of p38 acetylation and avoids p38-mTORC2-AKT overactivation, and SUMOylation-deficient Sirt2 loses its ability to inhibit tumor processes 134. Loss of *SIRT2* leads the endogenous FOXO1 acetylated in colon cancer by dissociating from SIRT2 and binding acetylated FOXO1 to the E1-like protein Atg7, which affects the autophagy process that leads to cell death and thus exerts an oncogenic effect 135. In a word, SIRT2 exerts tumor suppressive effects mainly by regulating cell cycle, DNA damage, cell proliferation, oxidative stress and autophagy **(Figure 4B, H)**.

#### SIRT3

The tumor suppressor effects of SIRT3 are primarily associated with metabolism regulation, and the regulation of cancer metabolic reprogramming by SIRT3 is mainly achieved through modulation of tumor cell glycolysis. In breast and gastric cancers, deletion of *SIRT3* promotes ROS production, leading to the stabilization of HIF-1α, a transcription factor controlling the expression of glycolytic genes. Besides, SIRT3 hydroxylates HIF-1α through prolyl hydroxylase (PHD) deacetylation, thereby controlling the expression of glycolytic genes, altering their stability, and attenuating their oncogenic effects 136. Furthermore, SIRT3 deacetylates and activates the mitochondrial pyruvate dehydrogenase complex (PDC), inhibiting glycolysis and promoting apoptosis in cancer cells 137. Moreover, SIRT3 deacetylates and inactivates Cyclophilin D (CYPD) and inhibits breast cancer glycolysis. SIRT3 also stabilizes p53 to inhibit glycolysis in wt-p53 cancer cells. From the mechanism of action, SIRT3 interacts with phosphatase and tensin homolog (PTEN) in the nucleus to modulate PTEN activity, thereby inhibiting Murine double minute 2 (MDM2) transcription, which is responsible for p53 degradation 138. Aspartic acid is an important amino acid for cell proliferation and can be regulated by GOT2. In addition to regulating the glycolytic pathway, SIRT3 deacetylated and inhibited GOT2, thereby impeding the formation of pancreatic tumors 139. Additionally, in hepatocellular cancer, pro-apoptotic protein BCL2-associated X protein (Bax) expression and mitochondrial translocation are induced by SIRT3 deacetylation and activation of glycogen synthase kinase-3β (GSK-3β) 140. The proto-oncogene product Skp2 is deacetylated and destabilized by SIRT3 in breast and prostate malignancies. Conversely, the acetylation of Skp2 is increased and its stability and cytoplasmic retention are enhanced upon SIRT3 inactivation, thereby promoting cell motility, proliferation, and carcinogenesis. Furthermore, cytoplasmic Skp2 increases cell migration through ubiquitination and E-calmodulin breakdown, while acetylation of Skp2 in the nuclear localization signal (NLS) encourages its cytoplasmic retention 141. Furthermore, SIRT3 deacetylates the K56 site of histone H3, which improves non-homologous end-joining repair of DNA and thus maintains genome integrity 142. Importantly, SIRT3 deacetylates the oncogene Lon protease-1 (LONP1), promotes ESCRT0 complex sorting and K63 ubiquitination, and reduces the energy supply of oxidative phosphorylation (OXPHOS), thereby inhibiting primary tumor growth **(Figure 4 C, H)** 143.

#### SIRT4

The role of SIRT4 in tumors is mainly related from the regulation of metabolism and genomic stability. *SIRT4* mRNA levels are reduced in human cancers such as pancreatic, gastric, ovarian, renal, prostate, liver, or lung cancers 144. In breast, colorectal 145, B-cell lymphoma and thyroid cancers, SIRT4 protein levels were significantly lower than their non-tumor tissue counterparts 146. In a mouse model of intestinal cancer, deficiency in *SIRT4* leads to dysregulated glutamine and nucleotide metabolism in intestinal adenomas, characterized by increased proliferation, enhanced glutamine uptake, and inhibition of slave nucleotide biosynthesis 147. By preventing the ADP-ribosylation of GDH, overexpression of *SIRT4* inhibited glutamate metabolism. This decreased the amount of energy and materials needed for the synthesis of proteins and nucleic acids and prevented the growth, migration, and invasion of tumor cells 148. By inhibiting GDH, SIRT4 also stops the cell cycle, provides sufficient time for gene repair, reduces the accumulation of DNA from cellular damage, maintains genomic stability, and thus reduces the occurrence of oncogenic mutations 149. The battle between SIRT4 and mammalian target of rapamycin protein complex 1 (mTORC1) is also interesting in the development of cancer. mTORC1 decreases SIRT4 protein levels by disrupting the cAMP-dependent transcription factor ATF-4 (CREB2). It also corresponds with the nutritional state and metabolism of cells. Conversely, reduced SIRT4 mRNA levels boost the expression of genes downstream of mTORC1, including *MYC, CCND1, HIF1A,* and* SREBP1*. The proliferation and survival of the prostate cancer cell line DU145 and the colon cancer cell line DLD1 depend on the reciprocal inhibition of SIRT4 and mTORC1 150. Similarly, the mTORC1-c-Myc pathway reconfigures methionine metabolism and facilitates the progression of HCC by suppressing SIRT4-mediated ADP ribosylation of MAT2A 151. Furthermore, in response to the stressful stimulus of folate deprivation, SIRT4 deacetylates the conserved lysine 50 (K50) residue in methylenetetrahydrofolate cyclic hydrolase 2 (MTHFD2), promotes Cullin 3 E3 ligase-mediated proteasomal degradation, and destabilizes MTHFD2. The aforementioned actions impede the synthesis of NAD phosphate and the accumulation of intracellular ROS in tumor cells, thereby suppressing the proliferation of breast cancer cells 152.

The regulation of mitochondria, cell cycle, and regulated cell death constitutes integral components of SIRT4 anti-cancer mechanism. In NSCLC, SIRT4 inhibits cancer cell proliferation, invasion, and migration via disruption of mitochondrial fission and suppression of the function of the mitochondrial fission-associated protein Drp1. The active form of phosphorylated Drp1 (S616) additionally contributes to the sequestration of Drp1 around the mitochondrial membrane and enhances the rate of mitochondrial fission 153. SIRT4 overexpression induces G1 arrest of the cell cycle by suppressing the expression of phosphorylated extracellular signal-regulated kinase phosphorylation of ERK (p-ERK), cell cycle protein D, and cell cycle protein E 149. Additionally, SIRT4 activates phosphorylation of the p53 protein by inhibiting glutamine metabolism, with involvement of AMPKα in the SIRT4-mediated regulation of autophagy and p53 phosphorylation. Moreover, the autophagic regulation of the SIRT4/AMPKα/p53 axis suppresses tumorigenesis and progression in pancreatic ductal adenocarcinoma **(Figure 4 D, H)** 154.

#### SIRT5

In pancreatic ductal adenocarcinoma (PDAC), SIRT5 is a key tumor suppressor. The primary mechanism by which SIRT5 exerts its anti-cancer effect is through the regulation of metabolic processes. The suppression of glutamine and glutathione metabolic pathways and the prevention of PDAC progression involve the down-regulation and deacetylation of aspartate aminotransferase GOT1 at the Lys369 site by SIRT5 155. Furthermore, SIRT5 increases ROS levels and NADP/NADPH ratios in GC cells, desuccinylates 2-oxoglutarate dehydrogenase (OGDH), downregulates its activity, decreases mitochondrial membrane potential (ΔΨm), an ATP product, and interferes with mitochondrial function and redox state to prevent GC cell growth and migration 156. In addition, enhanced SIRT5 activity leads to cell cycle arrest of tumor cells in G1/S phase due to negative regulation of cell cycle protein-dependent kinase 2 (CDK2) and inhibition of glycolysis 157. Moreover, SIRT5 deacetylates STAT3, blocking its ability to participate in the metabolism of mitochondrial pyruvate. In lung cancer A549 cells, down-regulation of SIRT5 causes STAT3 to be acetylated and translocated into the mitochondria. Besides, by interacting with the PDC, STAT3 speeds up the conversion of pyruvate to acetyl-coenzyme A, increasing the synthesis of ATP that supports cell growth 158. Furthermore, the SIRT5/p53 axis plays a critical role in preventing colon tumor growth, therefore attenuating the reprogramming of glycolytic metabolism and proliferation of intestinal epithelial cells **(Figure 4 E, H)** 159.

#### SIRT6

SIRT6 exerts a protective effect against tumor growth, enhances programmed cell death, and inhibits cellular proliferation in lung, colorectal, and ovarian cancers 160. The acetylation modification of histone H3K9ac, a hallmark of transcriptionally active promoters, plays a crucial regulatory role in the maintenance of DNA damage repair and telomere stability 161. The expression of HIF1α and MYC is suppressed by SIRT6 through deacetylation of H3K9 and K56 at the promoters of various glycolytic and ribosomal protein genes 162. Additionally, SIRT6 inhibits HIF-1α, which in turn regulates glycolytic regulators such as lactate dehydrogenase (LDH), glucose transporter-1 (GLUT1), pyruvate dehydrogenase kinase-1 (PDK1), and phosphofructokinase-1 (PFK1). Pyruvate kinase M2 (PKM2), a nuclear isoenzyme that boosts aerobic glycolysis and encourages tumor growth even in hypoxic environments, can inhibited by SIRT6 through deacetylation 163. In PDAC, downregulation of SIRT6 leads to hyperacetylation of H3K9 and H3K56 at the Lin28b (negative regulator of let-7 microRNA) gene promoter. This results in a looser chromatin state, which facilitates the expression of downstream let-7 target genes, oncoproteins like HMGA2, IGF2BP1, and IGF2BP3, and Myc transcription factor-driven expression. All of these processes support the growth and spread of PDAC 164. In addition, SIRT6 inhibits EMT and metastasis by suppressing Twist1 expression in NSCLC cells. Males absent on the first (MOF) -mediated acetylation of SIRT6, resulting in reduced SIRT6 deacetylase activity, impedes the interaction of SIRT6 with the transcription factor FOXA2 and leads to transcriptional activation of ZEB2, which ultimately attenuates the tumor-suppressor function of SIRT6 in NSCLC and promotes NSCLC progression 100. SIRT6 deficiency significantly promotes liver injury and HCC by inhibiting the ERK1/2 pathway **(Figure 4 F, H)** 165.

#### SIRT7

The anti-tumor effect of SIRT7 is mainly achieved through its remodeling of chromatin. Down-regulation of SIRT7 inhibition of E-Cadherin expression to promote EMT, thereby enhancing bladder cancer cell migration and invasion. This is caused by an acetylation mechanism and elevated total levels of the histone methyltransferase EZH2, which deposit the inhibitory histone mark H3K27me3 on the promoter of the CHD1 gene through EZH2-mediated means, hence suppressing the production of the gene 166. SIRT7 interacts with EST1 and its co-binding to the TEK promoter leads to H3K18ac deacetylation to inhibit its expression, which inhibits invasion and metastasis of breast cancer cells 167. Additionally, by encouraging SMAD4 deacetylation, SIRT7 prevents EMT in the metastasis of oral squamous cell carcinoma (OSCC) 168. SIRT7 lowers pro-caspase 3 activity and indirectly decreases the expression of pro-apoptotic proteins Bax and Bcl-2 during the progression of breast cancer tumors. Furthermore, CDK4/6, CyclinD1/2, and the G1/S and G2/M cell cycle checkpoints are all regulated by p38MAPK 169. However, as patients progress to more advanced metastatic stages, SIRT7 mRNA levels gradually decline. SIRT7 dysregulation is associated with breast cancer lung metastasis through activating TGF-β signaling and promoting EMT. SIRT7 hyperphosphorylation binds and stabilizes SKP2, restricting EGF-induced protein conversion and thereby inhibiting AKT activation 170. Interestingly, in breast cancer, *SIRT7* knockdown promotes breast cancer metastasis by leading to increased lamina-associated polypeptide 2 alpha (LAP2α) ubiquitination-dependent degradation and decreased LAP2 α protein levels leading to chromosomal instability **(Figure 4 G, H)** 171.

### Tumor- promoting

#### SIRT1

**Figure 5:**
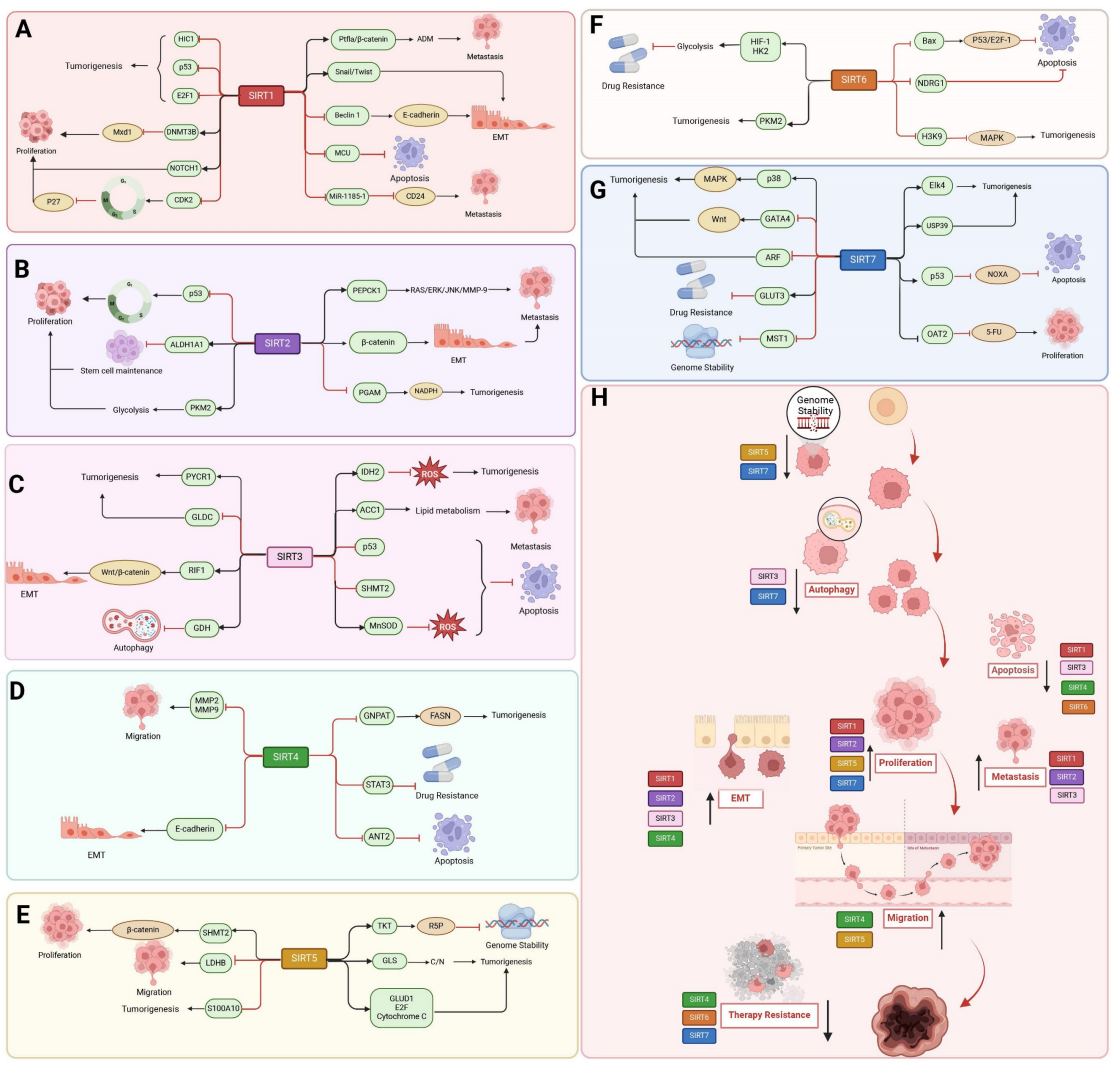
** The oncogenic role of SIRT1-7.** The SIRT1-7 enzymes can facilitate tumor progression by modulating various substrates involved in tumor initiation, development, cell cycle regulation, epithelial-mesenchymal transition (EMT), apoptosis, autophagy, metabolism, genomic stability maintenance, and response to cancer therapeutics.

The carcinogenic effect of SIRT1 is mainly manifested in its influence on tumor invasion and migration, and participates in the inhibition of programmed cell death. SIRT1 plays a function in increasing tumor formation in stomach, colon, prostate, and skin malignancies by having a higher expression level in these tumors 174. Through deacetylation modification and suppression of tumor suppressors, including E2F1, the tumor protein p53, and hypermethylation in cancer 1 (HIC1), SIRT1 promotes the formation of tumors. In pancreatic cancer, SIRT1 regulates adenohypophysis to ductal chemotaxis (ADM) through deacetylation of pancreatic transcription factor-1a and β-catenin, which enhances cell viability in pancreatic cancer, thereby promoting cell proliferation and tumor formation 175. By suppressing the promoters of the target genes, forkhead transcription factor 3 (FOXO3) and GRHL3, the SIRT1/CRL4B complex encourages the migratory and invasive capacity of pancreatic cancer cells 176. In melanoma cells, SIRT1 silences MXD dimerization protein 1 (Mxd1) by binding to DNMT3B, thereby increasing resistance to dead-cancer-induced stress, which leads to increased Myc activity and drives melanoma progression 177. Additionally, SIRT1 stimulates the activity of EMT regulators Snail and Twist or suppresses E-cadherin expression to facilitate EMT and metastasis in HCC and prostate cancer. By deacetylating Beclin 1, SIRT1 can also hasten the autophagic breakdown of E-cadherin in melanoma 178. Recently, another study demonstrated that in hematologic malignancies, SIRT1 exerts both oncogenic or oncostatic effects under different circumstances, depending on the disease type and oncogenic drivers. Indeed, SIRT1 was shown to promote leukemogenesis in CML and FLT3-ITD acute myeloid leukemia (AML). On the other hand, SIRT1 activation *in vivo* showed antiproliferative effects against MLL-rearranged leukemia and myelodysplastic syndrome. SIRT1 enhances NOTCH1-induced T-cell acute lymphoblastic leukemia (T-ALL) development and mediates resistance to NOTCH1 inhibition in a deacetylase-dependent mechanism 179. In addition, SIRT1 can also be upregulated by MYC in T-ALL and deacetylates CDK2 affecting the cell cycle and promoting phosphorylation and subsequent degradation of p27 180. In colon cancer, SIRT1 inhibition may be a therapeutic strategy. SIRT1 inhibition increases the acetylation of mitochondrial calcium uniporter (MCU), leading to mitochondrial Ca2+ overload and depolarization, and ultimately to apoptosis in colorectal cancer (CRC) cells 181. SIRT1 inhibits miR-1185-1 expression through histone deacetylation and targets the 3'UTR of CD24, thereby increasing stemness and invasiveness of CRC cells. Thus, the SIRT1-miR-1185-1-CD24 axis is thought to play an important role in regulating cancer stemness **(Figure 5 A, H)** 180.

#### SIRT2

SIRT2 has pro-cancer effects in numerous cancers, including breast, lung, gastric, liver, pancreatic, and colorectal cancers, where it induces tumor development mainly by altering the tumor microenvironment, inducing immune evasion, and regulating energy metabolism 182. SIRT2 is also pro-carcinogenic through deacetylation. In breast cancer, SIRT2 overexpression deacetylates p53, leading to its inactivation, impairing the regulation of G1/S and G2/M and allowing damaged cells to continue to proliferate 183. Additionally, acetyltransferase P300/CBP-associated factor (PCAF) and SIRT2 are in charge of controlling the acetylation status of ALDH1A1 K353. Lysine 353 (K353) acetylation suppresses ALDH1A1 activity. Breast cancer's stem cell number and ability to self-renew are inhibited by ALDH1A1 acetylation. On the other hand, NOTCH signaling promotes breast cancer stem cells by activating ALDH1A1 through the induction of SIRT2, which results in ALDH1A1 deacetylation and enzyme activation 184. Furthermore, PKM2 modulation can be used by breast cancer cells to rewire their glycolytic metabolism when SIRT2 function is lost 185. Phosphoglycerate amphoteric acid mutase (PGAM), a glycolytic enzyme, is crucial for balancing the production of energy with the generation of less power and for the biosynthesis of amino acids and nucleotide precursors. Small RNAi or small compounds that inhibit PGAM reduce tumor development and cell proliferation. In NSCLC, deacetylation of PGAM-K100 by SIRT2 leads to increased NADPH generation and faster tumor growth 186. The pro-carcinogenic effects of SIRT2 are closely related to metabolic processes in cancer. For instance, SIRT2 activates the RAS/ERK/JNK/MMP-9 pathway in gastric cancer to raise PEPCK1 protein levels, mitochondrial activity, and cell migration and invasion. Furthermore, RAS triggers the activation of ERK and JNK, two significant downstream MAPCs. The ERK/JNK pathway is also responsible for the up-regulation of MMP-9, which is perhaps one of the most important molecules in the spread of cancer 187. Additionally, SIRT2 is an important tumor promoter in hepatocellular cancer. By focusing on the protein kinase B/glycogen synthase kinase-3β/β-catenin signaling pathway, which is involved in HCC cellular motility and invasiveness, SIRT2 overexpression stimulates EMT and motility of HCC cells **(Figure 5 B, H)** 188.

#### SIRT3

The oncogenic role of SIRT3 is mainly related to the metabolic dependence mode and ROS preference degree of different cancers. In malignant B cell lines, SIRT3 expression is reduced. This lower expression level correlates with hyperacetylation of isocitrate dehydrogenase 2 (IDH2) and superoxide dismutase 2 (SOD2) mitochondrial proteins, reduced enzyme activity, and elevated ROS levels 189. SIRT3 promotes lipid metabolism in cervical cancer cells by deacetylating acetyl coenzyme A carboxylase (ACC1). The migration and invasion of cancer are aided by this reprogramming of fatty acid metabolism 190. In addition, SIRT3 is deacetylated in PTEN-deficient NSCLC and promotes p53 degradation, thereby exerting a pro-oncogenic effect 191. Through the action of serine hydroxymethyltransferase 2 (SHMT2) at the Lys95 site, SIRT3 deacetylates serine and glycine and prevents its lysosomal-dependent degradation, hence accelerating the development of colorectal cancer 192. Similarly, SIRT3 deacetylates and activates pyrroline-5-carboxylic acid reductase 1 (PYCR1), which is involved in proline synthesis and promotes cell proliferation by catalyzing the reduction of P5C to proline and the simultaneous production of NAD and NADP 193. In addition, SIRT3 promotes glycine decarboxylase (GLDC) K514 deacetylation, which triggers K33-linked polyubiquitination of the ubiquitin ligase NF-X1 at the K544 site, leading to its degradation via the proteasomal pathway, and thus inhibits glycine catabolism, pyrimidine synthesis and glioma tumorigenesis 194. The mitochondrial superoxide dismutase 2 (MnSOD), which can convert superoxide anion to H_2_O_2_, remains deacetylated in CLL cells due to SIRT3 overexpression. SIRT3 overexpression in chronic lymphocytic leukemia (CLL) prevents ROS overaccumulation and reduces detrimental cytotoxic effects 195. SIRT3 has the ability to act as an oncogene in some tumors where OXPHOS continues to be the predominant source of energy. In chronic lymphocytic leukemia cells, SIRT3 regulates fatty acid oxidation, which is essential for OXPHOS and ATP synthesis, hence promoting cancer cell survival. SIRT3 promotes the growth of tumors in glioblastomas and preserves stemness in glioma stem cells by improving mitochondrial activity that affects metabolism 196. Furthermore, SIRT3 regulates the deacetylation of replication timing regulator 1 (RIF1) to activate the Wnt/β-catenin pathway, which promotes stemness, metastasis, and EMT in NSCLC 197. According to reports SIRT3 also affects the role of the TCA cycle thereby promoting cancer development. SIRT3 depletion hampers glutamine flow to the TCA cycle through decreasing glutamate dehydrogenase (GDH) and acetyl coenzyme A pools, which triggers autophagy and death of Diffuse large B cell lymphomas (DLBCLs) **(Figure 5 C, H)** 198.

#### SIRT4

Most studies have investigated the tumor suppressor function of SIRT4, but some studies have also confirmed that SIRT4 is also pro-carcinogenic, this is also related to the metabolic preferences of different cancers. Tissue studies of esophageal cancer and adjacent non-tumor tissues have revealed that the protein level of SIRT in esophageal cancer tissues is significantly higher than that in normal paraneoplastic tissues, and that the survival time of patients with high SIRT4 expression is shorter than that of patients with low SIRT4 expression 199. While acetyl-CoA acetyltransferase 1 (ACAT1), a mitochondrial enzyme that acetylates GNPAT at the K128 site, promotes lipid metabolism and hepatocarcinogenesis by stabilizing fatty acid synthase (FASN) metabolism and hepatocarcinogenesis, SIRT4 deacetylates glycerol O-acyltransferase (GNPAT) and decreases its expression in HCC 200. In addition, SIRT4 prevents the accumulation of DNA damage and reduces cell death resulting from DNA damage, but under extreme conditions of DNA damage, such as in the presence of chemotherapeutic agents, SIRT4 protects tumor cells and thus acts as an oncogene 199. Overexpression of the target genes of the STAT3 signaling system, *MYC* and *CCND1*, confers tamoxifen resistance in ER-positive breast cancer cells. SIRT4 also suppresses the phosphorylation and nuclear translocation of tyrosine 705 (Y705) in STAT3, hence blocking the STAT3 signaling pathway 201. In addition, the PAK6-SIRT4-ANT2 complex inhibited apoptosis in prostate cancer cells, and SIRT4 deprived ANT2 acetylation at the K105 site and promoted ubiquitinated degradation of ANT2. In this process, PAK6 regulated the level of acetylation of ANT2 and modulated its stability through the SIRT4 92. Furthermore, SIRT4 minimizes the accumulation of DNA damage and decreases cell death caused by DNA damage; nevertheless, under extreme conditions of DNA damage, such as in the presence of chemotherapy treatment, SIRT4 protects tumor cells and thereby operates as an oncogene **(Figure 5 D, H)** 199.

#### SIRT5

The carcinogenic effect of SIRT5 is primarily manifested through its involvement in the regulation of DNA damage, metabolism, and programmed cell death. In colorectal cancer, high expression of SIRT5 regulates the non-oxidative pentose phosphate pathway by activating transketolase (TKT) in a depropionylation-dependent manner, which increases ribulose-5-phosphate (R5P) production and supports nucleotide synthesis, thereby enhancing DNA damage and cell proliferation in CRC 202. Desuccinylation of residue K164 by SIRT5 protects the mitochondrial enzyme glutaminase (GLS) from K158 ubiquitination and subsequent degradation, and increases carbon and/or nitrogen levels, thereby promoting breast tumorigenesis 203. In CRC cells, SIRT5 promotes colorectal carcinogenesis by enhancing glutamine catabolism through activation of glutamate dehydrogenase 1 (GLUD1) in a deglutecosylation-dependent manner 204. Furthermore, by desuccinylating serine SHMT2, which catalyzes the catabolism of serine and provides methyl for cellular methylation events through single-carbon metabolism, SIRT5 also contributes to the proliferation of tumor cells 205.

Notably, SIRT5 can also promote cancer development by regulating proliferation and migration through deacetylation of its substrates. SIRT5 can mediate the deacetylation of lactate dehydrogenase B (LDHB) thus promoting autophagy and tumorigenesis in colorectal cancer 16. SIRT5 promotes HCC proliferation and invasion by targeting the transcription factor E2F1. Similarly, SIRT5 facilitates the degradation of S100A10 protein, thereby promoting invasive migration in gastric cancer 16. SIRT5 also promotes HCC growth by inhibiting apoptosis through deacetylation of cytochrome C. SIRT5 also inhibits apoptosis through deacetylation of cytochrome C. SIRT5 can also inhibit apoptosis through deacetylation of cytochrome C 206. Moreover, SIRT5 knockdown disrupts the production of ribulose-5-phosphate, which is essential for nucleotide synthesis, leading to persistent and irreparable DNA damage in CRC cells, which results in cell cycle arrest and enhanced apoptosis **(Figure 5 E, H)** 202.

#### SIRT6

Most data support a role for SIRT6 as a tumor suppressor, but some evidence suggests that SIRT6 may promote tumor survival in an environmentally dependent manner. For example, resistance to programmed cell death and DNA damage are important contributors to SIRT6 cancer promotion. SIRT6 is carcinogenic in prostate cancer, skin cancer and breast cancer 173. SIRT6 deacetylates H3K9 in HCC cells, which prevents Bax transcription. Consequently, it increases p53 and E2F-1 chromatin accessibility, which prevents apoptosis 207. Overexpression of SIRT6 is required for the induction of transforming growth factor (TGF)-β1 and H_2_O_2_/HOCl-activated ROS-mediated tumorigenesis. TGF-β1 upregulates SIRT6 expression, induces activation of ERK and Smad pathways, and alters the effects of these proteins on cellular senescence 208. Furthermore, in multiple myeloma (MM) cells, overexpressed SIRT6 interacts with genes related to the transcription factors ELK1 and ERK signaling. By binding to the promoter of the MAPK pathway genes and de-acetylating H3K9, SIRT6 downregulates the expression of these genes, promoting cell proliferation and providing resistance to DNA damage 209. Recently, another study demonstrated that SIRT6 protein levels are negatively correlated with anoikisa, a form of apoptosis significant to cancer metastasis, and mechanistically SIRT6 inhibits apoptosis in by down-regulating the transcription of N-myc downstream regulated gene 1 (NDRG1) which is a negative regulator of the AKT signaling pathway 210. PKM2 is a nuclear isoenzyme that promotes aerobic glycolysis and tumor growth even in hypoxic environments. SIRT6 binds to and deacetylates nuclear PKM2 at lysine residue 433, leading to nuclear export of PKM2, which in turn leads to reduced cell proliferation, migratory potential, and invasiveness 163. Interestingly, studies revealed that SIRT6 increases glycolysis through the HIF-1α/HK2 signaling axis in drug-resistant cells and diminishes sensitivity to erlotinib in NSCLC cells **(Figure 5 F, H)** 211.

#### SIRT7

SIRT7 is overexpressed in invasive breast cancer, esophageal cancer, renal cell carcinoma, clear cell renal cell carcinoma, lung adenocarcinoma, prostate adenocarcinoma, HCC, thyroid carcinoma, cholangiocarcinoma, uterine corpus cancer, and gastric adenocarcinoma.^166^ SIRT7 promotes cancer cell proliferation and metabolism primarily by promoting ribosome biogenesis. SIRT7 also contributes to the adaptation of cancer cells to various stress conditions such as hypoxia, nutrient deprivation, oxidative stress, and chemotherapeutic drug exposure. In addition, SIRT7 exerts epigenetic control over the expression of specific genes involved in cancer progression, further contributing to the malignant phenotype 167. For instance, in HCC, USP39 interacts with and is deacetylated by SIRT7 and promotes its stability, thereby accelerating HCC cell proliferation and tumorigenesis 212. Through the deacetylation of H3K18ac in local promoters, SIRT7 and Elk4 work together to sustain oncogenic transformation and tumor growth 38. SIRT7 also interacts with p53, decreasing its binding to the NOXA promoter and reducing its transcription, effectively suppressing adriamycin-induced apoptosis. Similarly, SIRT7 decreases H3K18ac levels and downregulates the expression of the organic anion transporter protein OAT2 promoter, thereby decreasing 5-fluorouracil (5-FU) uptake 17. SIRT7 inhibits the transcriptional activity of GATA4 by promoting deacetylation of GATA4 and activates the Wnt signaling pathway to promote the development of ovarian cancer (OC) 213. In addition, SIRT7 inhibits MST1 through transcriptional regulation and post-transcriptional modification, thereby promoting YAP nuclear localization and transcriptional activation in HCC 214.

Interestingly, In NSCLC, SIRT7 directly interacts with tumor suppressor alternative reading frame (ARF) and prevents ARF from binding to nuclear phosphoproteins, thereby promoting proteasome-dependent ARF degradation and ultimately increasing the expression of tumorigenic genes 215. Studies have shown that SIRT7 can also affect drug sensitivity. In contrast, in breast cancer, SIRT7 was significantly upregulated in the early stages but gradually decreased with tumor progression 216. SIRT7 is involved in breast cancer proliferation, tumor growth and chemotherapeutic drug resistance through activation of the p38 mitogen-activated protein kinase (MAPK) pathway 167. SIRT7 can regulate the generation of GLUT3, which can transport gemcitabine in pancreatic cancer cells, by binding to its enhancer and modifying the quantity of H3K122 succinylation, therefore affecting the susceptibility of PC cells to gemcitabine **(Figure 5 G, H)** 217.

Taken together, the epigenetic and non-epigenetic effects of SIRTs constitute a complex and elegant network that regulates the different processes of cancer, showing different effects in different environments. Clearly, SIRTs are important regulators of metabolism and genome maintenance, and their ability to suppress tumor formation is well documented. However, there have also been reported instances of SIRTs-driven tumor survival that are influenced by cellular and environmental factors. The intricate functions of SIRTs in tumor progression and viability are still being assessed. In the context of healthy cells, DNA repair and genomic stability can prevent tumorigenesis; however, following specific types of transformation, these same functions can protect aberrant cells from genotoxic therapies or the accumulation of excess deleterious mutations. Interestingly, due to the different subcellular localization of SIRTs, SIRT1 and SIRT2 are localized in the nucleus and cytoplasm of the cell, and play diverse functions in cancer. SIRT3, SIRT4 and SIRT5 are localized in the mitochondria, and play different pro- and anti-tumorigenic roles in mitochondrial metabolism, including the TCA cycle, amino acid metabolism, fatty acid oxidation of the urea cycle, OXPHOS. SIRT6 and SIRT7 are localized only in the nucleus and mainly regulate genetic and metabolic activities. This dual role appears to be controversial, suggesting that SIRTs may be involved in tumorigenesis through different mechanisms under different cellular or systemic conditions. The functional balance between anticancer and pro-tumorigenic pathways may determine the final functional phenotype of SIRTs. The context-dependent role of SIRTs as tumor suppressors or tumor drivers opens the door to informed disease-specific pharmacological activation or inhibition. Similarly, the development of targeted SIRTs regulators will help to study SIRTs activity in different disease contexts 219. This also provides the possibility for the properly regulation of SIRTs with small molecule compounds under different conditions to achieve precise treatment of specific tumors.

## Targeting SIRTs with small-molecule modulators in cancer therapy

**Figure 6:**
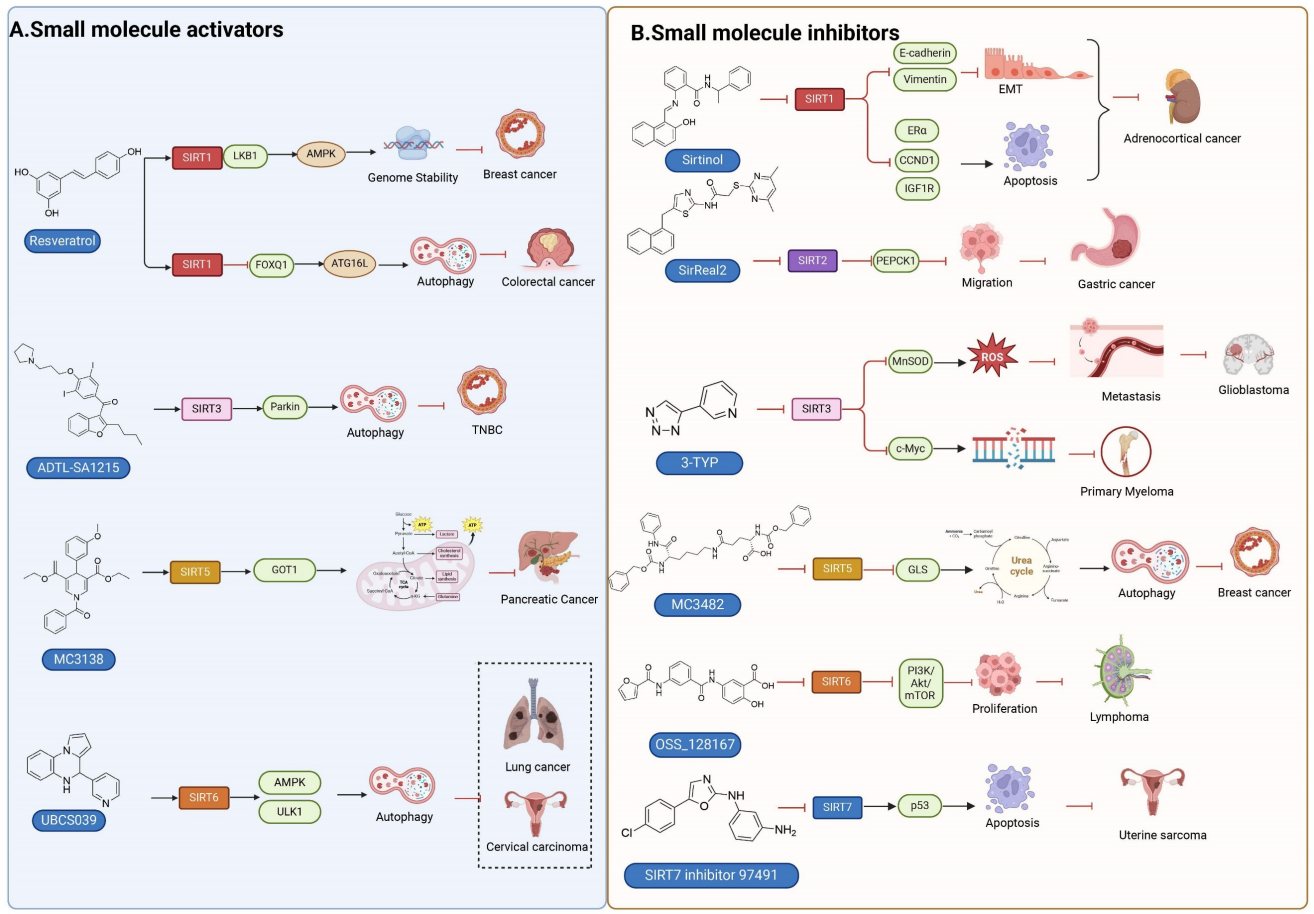
Mechanism of action of representative small molecule modulators targeting SIRTs.

The conventional treatments for cancer, including surgery, radiotherapy, and chemotherapy, have shown limited efficacy. Therefore, there is an urgent need to discover innovative drugs targeting cancer. Epigenetic alterations are a characteristic feature of cancer, and epigenetic enzymes play a dynamic and reversible role in controlling protein translational modifications rather than globally affecting the DNA sequence 220. Small molecule inhibitors and activators that targeting SIRTs are emblematic of the increasing number of anticancer medications being discovered and brought to the market that target epigenetic enzymes. Patients have responded better to the clinical efficacy and tolerance of epigenetic medications (**Figure 6, Table 5 and Table 6**) 221.

### Small-molecule modulators targeting SIRTs in preclinical cancer therapy

#### Activators

Natural products have long been used as medicines for the treatment of various human diseases. Lead compounds discovered from natural sources are used as novel templates for the development of more effective and safer drugs. Natural products produce biological activity by binding to biomolecules because natural products complement protein binding sites and because natural product-protein interactions have been optimized in nature. The natural phytochemicals' molecules can serve as pharmaceutical agents for the modulation of SIRT activity in cancer treatment 222. For instance, **Resveratrol**(3,5,4'-trihydroxystilbene) can activate several SIRTs. Resveratrol is a polyphenolic plant antitoxin, which was first isolated and characterized in white quinoa in 1939 223. Resveratrol deacetylates SIRT1 and activates hepatic Liver Kinase B1 (LKB1), leading to an increase in AMPK activity, which stimulates energy catabolism and improves cellular NAD^+^ levels, resulting in cancer inhibition 224. In addition, Resveratrol inhibits colorectal cancer progression by activating SIRT1, inhibiting the expression of the transcription factor FOXQ1, and controlling the regulatory promoter region of the 3′-UTR of the key autophagy molecule ATG16L, which triggers autophagy-related apoptosis 225. In HCC, Resveratrol inhibits mito-COX-2 acetylation, leading to a decrease in cell proliferation and mitochondrial fission, which occurs through up-regulation of SIRT3 226. In colorectal cancer, SIRT5 (resveratrol and sulforaphane) acts on the chemotherapeutic drug 5-FU, affecting the proliferation of cancer cells 227. SIRT7 expression is decreased in breast cancer cells and correlates with poor patient prognosis, and SIRT7 can affect the invasive ability of breast cancer cells by deacetylating and degrading Smad4. Resveratrol promotes SIRT7 deacetylation, which inhibits tumor cell invasion and survival 228**.**

**Quercetin** (3,5,7,3′,4′-pentahydroxyflavone) is a natural flavonoid found in fruits and vegetables in the form of glycosides 229. Quercetin activation of SIRT1 to induce autophagy in A549 and H1299 human lung cancer cells and contributes to lung cancer cell apoptosis 230. Recent studies have shown that quercetin targets not only SIRT1 but also SIRT5 to induce apoptosis in NSCLC cells. Mechanistically, quercetin binds to SIRT5 and inhibits the phosphorylation of PI3K/AKT through the interaction between SIRT5 and PI3K, which inhibits the repair process of HR and NHEJ and promotes apoptosis of NSCLC cells 231. In another SIRT6 assay performed using high performance liquid chromatography (HPLC), quercetin boosted SIRT6 activity. To initiate SIRT6-induced deacetylation, quercetin binds to acyl-binding channels that are selective for SIRT6. Furthermore, the substrate acyl-binding channel for SIRT6 is provided by a particular tiny Zn^2+^-binding domain and a cofactor-binding loop. With three hydrogen-bond donors and one hydrogen-bond acceptor, the SIRT6 protein provides a tentative pharmacophore of quercetin-binding sites. In the deacetylated genes H3K18ac and H3K9ac, quercetin activates SIRT6.One possible target for anticancer therapy is the H3K18ac gene 232.

In recent years, numerous small molecules capable of modulating SIRT1 activity have been identified and characterized utilizing a variety of methods, including high-throughput screening (HTS), computer- or fragment-based screening, focused library screening in conjunction with in-depth conformational studies, and *in vitro* and *in vivo* testing. The goal of recent research has been to find novel synthetic SIRT1 activating chemicals (STACs). **SRT1720** is a selective SIRT1 activator that discovered in 2007. In MM cells, SRT1720 promotes apoptosis through the SIRT1-AMPK signaling pathway 233. Similarly, SRT1720 inhibits NSCLC via the SIRT1-AMPK pathway, and these effects can be blocked by administration of the AMPK inhibitor Compound C. The inhibitor is also known to inhibit the SIRT1-AMPK signaling pathway. Notably, this hypoxic inactivation of the SIRT1-AMPK pathway leads to cisplatin and doxorubicin resistance 234. Activation of SIRT1 by SIRT1720 increased the expression of ZEB1 and vimentin, decreased the expression of E-cadherin, and promoted ferroptosis in HNC cells. Interestingly, resveratrol also exerts ferroptosis effects targeting SIRT1 235. SRT1460 uses an aminomethylcoumarin (AMC)-labeled peptide as a substrate and activates SIRT1 through a peptide KM-lowering mechanism that is similar to that of resveratrol 236. The SIRT1 agonists SRT1720 and SRT1460 are more effective in pancreatic cancer cells. However, SRT3025 has a substantial inhibitory effect on Panc-1 tumors *in vivo*. In addition STACs enhance the sensitivity of pancreatic cells to gemcitabine and paclitaxel in conjunction with other chemotherapeutic drugs 237.

Measurement of the reaction catalyzed by coupled SIRT1 and nicotinamidase (NMase) correlates SIRT1 deacetylase activity with ammonia production. A promising novel SIRT1 activator (SCIC2.1) was identified and characterized by HTS. Molecular modeling studies suggest that the NTD/SCIC2 recognition event should trigger the stabilization of the SIRT1 closed/activated conformation, which is more favorable for enzyme catalysis 238. Prolonged glucose restriction causes **SCIC2.1** to activate SIRT1 by lysine 382 site phosphorylation of p53, which causes metabolic reprogramming in stem cell cancer. The expression of SIRT1 was elevated by SCIC2.1, and this in turn affected the AMPK-p53-PGC1α signaling pathway to reduce metabolic stress and increase cell survival. Furthermore, in cells cultured under glucose-deprivation conditions, SCIC2.1 was able to govern mitochondrial activity in a time-dependent manner by reducing SOD2 expression and regulating SIRT3 expression 218.

By superimposing conformational and predicted binding pockets between SIRT1, SIRT2, SIRT3, and SIRT5, four different SIRTs have different distributions on Pocket L and Pocket U. The four SIRTs have a different distribution on Pocket L and Pocket U. The four SIRT3 activators have a different distribution on Pocket L and Pocket U. Therefore, the design of small molecule activators of SIRT3 targeting Pocket L or Pocket U led to the discovery of the targeting SIRT3 activator compound **ADTL-SA1215** by structure-guided design and high-throughput screening. Thus, ADTL-SA1215 activates SIRT3 and leads to SIRT3-driven autophagy/mitochondrial autophagy-associated cell death in triple-negative breast cancer cells, with significant antiproliferative and antimigratory activities. *In vitro*, ADTL-SA1215 significantly increased the expression of mitochondrial autophagy-related proteins PINK1, Parkin, ULK1, Ambra1 and Beclin1. That is, mitochondrial autophagy was induced through the SIRT3-Parkin pathway. In addition, ADTL-SA1215 exerted anti-tumor effects by targeting SIRT3-regulated autophagy in a TNBC mouse xenograft model 239.

The latest reports also have activators targeting SIRT5. A library of 1,4-dihydropyridine (1,4-DHP) compounds was synthesized based on structural similarity to the Sirtuin ligands NAM and 2-anilinobenzamide. The compounds of DHP enable subtype-specific SIRTs activation and scaffold derivatives specific for SIRT3 and SIRT5 240. **MC3138** is a DHP analog that replicates the effects of SIRT5 overexpression, inhibiting GOT1 enzyme activity by deacetylating GOT1 at the lysine 369 site, thereby decreasing PDAC cell viability. Additionally, MC3138 combined with gemcitabine showed synergistic benefits in CFPAC-1 and Colo357/FG cells, and this come-fit medication may be a potential therapeutic alternative for SIRT5 low-expressing PDAC 155. Interestingly, MC3138 modulates autophagy and reduces cancer cell viability. MC3138 activates SIRT5, which decreases the levels of metabolites in the glutamine, glutathione, and pyrimidine pathways, and triggers mitochondrial autophagy, which increases total and mitochondrial ROS to inhibit cancer cell proliferation 241.

**Anthocyanidins** are colored flavonoids, water-soluble pigments found in the plant kingdom and secondary plant metabolites 242. The most potent compounds of the anthocyanin group, anthocyanins, significantly enhance the deacetylation activity of SIRT6. Cell culture and *in vivo* studies on anthocyanins have shown that they possess anticarcinogenic properties against colon, skin and lung cancers. In addition, anthocyanins affect the expression levels of SIRT6-related genes such as Twist1 and GLUT1 243.

**UBCS039** is a synthetic pyrrolo[1,2-a]quinoxaline derivative, the first specific activator synthesized by SIRT6 244. Biochemical analysis reveals that the substrate-independent chemicals attach to the SIRT6 catalytic core, activating SIRT6-dependent deacetylation of peptide substrates and intact nucleosomes. UBCS039 binds directly to the catalytic core of SIRT6 and can effectively activate SIRT6 to target histone H3 deacetylation. UBCS039 binds to the SIRT6-specific acyl channel pocket and can induce autophagy in cancer cells 245. Activation of SIRT6 activity by BCS039 induces deacetylation of SIRT6 targeting the histone H3 site. In addition, SIRT6 targets SIRT6 to activate ROS, which mediates the activation of the AMPK/ULK1 pathway, thereby promoting phagocytosis 245.

**MDL-811** is a HDAC6 activator, and it markedly increased SIRT6 histone H3 deacetylation (H3K9Ac, H3K18Ac, and H3K56Ac) and suppressed the growth of several colon cancer cell lines. Cell lines and xenografts produced from patients were also used to establish MDL-811's anticancer activity *in vivo* 221. SIRT6 is commonly down-regulated in NSCLC and is associated with tumorigenes. To prevent tumor growth, **MDL-800** boosted SIRT6 deacetylase activity while decreasing phosphorylation of MEK and ERK (p-MEK and p-ERK). The antiproliferative effect of MDL-800 was significantly diminished by the SIRT6 knockdown was significantly attenuated, suggesting that MDL-800 is an effective activator to inhibit the proliferation of NSCLC 246.

#### Inhibitors

Nicotinamide (NAM) is an endogenous inhibitor of all SIRT isoforms, with IC_50_ values in the mid micromolar range. The mechanism of inhibition involves post-release recombination with the C pocket and successive nucleophilic attack of the pyridine nitrogen on the O-alkylimidate intermediate. NAM is the amide derivative of niacin (vitamin B3 or PP). It is a precursor to the nicotinamide-adenine dinucleotide NAD 251. NAM inhibits both SIRT1 and SIRT2 activity. Nicotinamide promotes NAD^+^ redox homeostasis and provides NAD^+^ as a substrate for the SIRT family of proteins, which cleave NAD^+^ to produce NAM while deacetylating the target protein. Once NAM enters the cell, it will be rapidly converted to NAD^+^, so the concentration of NAM in the cell will decrease rapidly, while the concentration of NAD^+^ will increase. Therefore, the activity of SIRT1 will be inhibited for a limited period of time and then enhanced 252. NAM is used as a potent SIRT2 inhibitor with EC_50_ values comparable to other inhibitors such as sirReal2 and thiomyristoyl 248,249. 5-20 mM NAM induces mitochondrial autophagy, and its mechanism of action through increasing NAD content. At doses up to 5 mM, NAM demonstrates cytoprotective activity that increased the viability and replicative potential of cultured cells, whereas at doses greater than 20 mM, it led to apoptotic cell death 254.

Additionally, **sirtinol** is a synthetic inhibitor specific for SIRT1 and SIRT2 255. Sirtinol induces senescence-associated β-galactosidase activity and expression of fibrinogen activator inhibitor 1 in breast cancer and lung cancer. It promotes the responsiveness of extracellular signal-regulated kinases, c-jun N-terminal kinase and p38 MAPK to epidermal growth factor (EGF) and insulin-like growth factor-I (IGF-I) 256. In the breast cancer MCF-7 cell line, sirtinol reduced SIRT1 expression and successfully promoted cell death, resulting in G1 phase cell cycle arrest and apoptotic cell death, as well as autophagic cell death 257. In breast cancer, sirtinol inhibits the proliferation of breast cancer cells and stimulates them to initiate the cell death process. Sirtinol inhibits SIRT1 more efficiently than SIRT2. Sirtinol interacts with SIRT1 through hydrogen bonding with Gln345 and His363 residues, while it interacts with SIRT2 through hydrogen bonding with Gln167. Sirtinol inhibits the proliferation of breast cancer cells and stimulates them to initiate cell death. Sirtinol helps to increase the acetylation of the lysine residue at position 382 of the p53 protein, leading to its activation 258. Recent reports have also shown that sirtinol inhibits SIRT1, downregulates several proteins such as ERα, CCND1, and IGF1R to interfere with the E2/ERα and IGF1R pathways and activate apoptosis, thereby reducing the growth of H295R and SW13 adrenocortical carcinoma cells 259.

**Salermide** is an analog of the 2-hydroxynaphthalene derivative sirtinol, i.e., it changes the amide portion carried by sirtinol to a reverse amide and ultimately moves the amide side chain from the 2' to the 3' position of the benzene ring. The key chemical features of this function are in different spatial orientations, and salermide retains the polar interaction: a hydrogen bridge between the oxygen and Q167 side chains of the amide, which is strongly inhibitory to SIRT1 and SIRT2 *in vitro* 260. It induces substantial apoptosis in cancer but not in untransformed cultured cells, including cancer stem cells from colorectal cancer and glioblastoma multiforme (GBM) 260. Salermide triggers p53-dependent cancer-selective apoptosis by activating aberrantly suppressed pro-apoptotic genes in cancer cells through SIRT1-mediated K16H4 deacetylation 261.

**Selisistat (EX-527 or SEN0014196)** is a SIRT1 inhibitor that was first discovered in 2005 and was the first sub-micromolar SIRT1 inhibitor described with cell permeability 262. Selisistat interacts with the C pocket of SIRT1 and neighboring hydrophobic regions. Furthermore, selisistat binds with SIRT1 following the formation of the alkyl acyl ester intermediate. This binding blocks the release of 2'-O-acetyl-ADP-ribose and establishes a persistent inhibitory complex with SIRT1 263. Selisistat was later found to also inhibit SIRT2, but to a lesser extent. Selisistat blocks protein-protein interactions between breast cancer deletion 1 (DBC1) and SIRT1 through an acetylation-independent mechanism. An endogenous protein called DBC1 binds to SIRT1 and prevents its catalytic function. When combined with HSP-90 inhibitors in cancer stem cell-like cells, selenistat exhibits less cytotoxicity than the latter. Cytotoxicity was reduced by the inhibitor. In addition, 1 μM selisistat inhibited the clonogenic ability of ovarian cancer cells with or without SIRT1 overexpression. At 600 nM, it inhibited cell migration and suppressed EMT in chemoresistant esophageal cancer cells 264. By blocking SIRT1, selisistat prevents cervical cancer by restoring transcriptionally active K382-acetylated p53 in HPV cell lines. This arrests the G0/G1 cell cycle and prevents HPV cells from proliferating. Furthermore, HPV cells were more sensitive to sublethal dosages of common genotoxic medications when selisistat was used 265.

**Cambinol** is a 2-hydroxynaphthalene derivative with SIRT1 and SIRT2 inhibitory effects, which inhibits the NAD^+^-dependent deacetylase activity of SIRT1 and SIRT2. Cambinol acts on lymphoma cells to induce apoptosis by promoting the hyperacetylation of p53 and the transcriptional repressor protein BCL6. In addition, cambinol-induced SIRT1 inhibition sensitized the cells to chemotherapeutic agents 266.

**Tenovin-1** and **tenovin-6** were discovered as possible nicotinamide analog deacetylase inhibitors by yeast genetic screens, biochemical experiments, and target validation investigations in mammalian cells. Tenovins increases p53 protein levels, which is consistent with SIRT1 inhibition, while decreasing the lifespan of several cancer cell lines, implying that acetylation retention promotes p53 protein stability. In contrast, kinetic investigations into tenovins-mediated sirtuin inhibition suggest a noncompetitive mechanism for both acyl lysine substrates and NAD co-substrates, which is comparable to nicotinamide-mediated SIRT inhibition 267. It has been shown that tenovin-1 and tenovin-6 act by inhibiting the protein deacetylation activity of SIRT1 and SIRT2, leading to a decrease in the expression of the mitotic checkpoint regulators BUB3, BUB1, and BUBR1, which is capable of slowing down the growth of tumors from highly aggressive melanoma cell lines 268. In Ewing's sarcoma, early gene expression alterations and DNA damage in Bcl-2 family members following treatment with tenovin-1 led to the release of MOMP and AIF, which in turn led to mitochondrial dysfunction and ROS accumulation, ultimately leading to cell death 269.

**SPC-180002** is a novel SIRT1/3 dual inhibitor with antiproliferative effects on a wide range of cancer cells by impairing mitochondrial function and redox homeostasis. SPC-180002 is an attractive new anticancer drug candidate as it significantly impedes tumor progression in mice treated with it 270. Furthermore, considering the recent reports of SIRT1 modulation of chemosensitivity to doxorubicin and cisplatin, SPC-180002 may have potential for combination therapy studies with DNA damaging agents 271.

A novel SIRT2 inhibitor, **AGK2**, was identified in the pathogenesis of Parkinson's disease (PD), and AGK 2 differed from Sirtinol, an inhibitor of SIRT2, by a significant up-regulation of acetylation modifications and an increase in the level of acetylated microtubule proteins 272. 2-Cyano-3-[5-(2,5-dichlorophenyl)-2-furanyl]-N-5-quinolinyl-2-propenamide (AGK2) is a strong and potent inhibitor of SIRT2, but has less effect on SIRT1 and SIRT3.^273^ In HCC, AGK2 increases fibrinogen-like protein 1 (FGL1) acetylation *in vitro* while decreasing FGL1 protein levels, which are immune checkpoint ligands. In mice, blocking both AGK2 and programmed death ligand 1 (PD-L1) substantially suppress tumor growth and enhance overall survival 274. By suppressing the expression of cell cycle proteins D1, CDK4, and CDK6, as well as preventing HeLa cervical cell proliferation and colony formation, AGK2 also mediates cell cycle arrest in the G1 phase 275.

**Inauhzin (INZ)** is a computational structure-based screened compound that promotes p53-dependent apoptosis in human cancer cells by inhibiting SIRT1 activity and inducing p53 acetylation without causing significant genotoxic stress. The compound inhibited the growth of xenograft tumors derived from p53-containing lung and colon cancer cell lines, but had minimal effect on tumors derived from p53-inactive HCT116 cells 276. By simultaneously targeting SIRT1 and inosine monophosphate dehydrogenase 2, INZ inhibits the development of cancer cells by inducing ribosomal stress, RPL11/RPL5-MDM2 association, activating p53, and blocking SIRT1 277.

**SirReal2** attaches to residues in a particular pocket near the zinc-binding domain, causing SIRT2 to open up and undertake ligand-induced conformational changes in the active site. In cells of lung, colorectal, breast, lymphoma, and cervical cancer, SirReal2 exhibits antiproliferative properties. In HeLa cells, SirReal2 leads to α-microtubulin and microtubule hyperacetylation, but does not disrupt the cell cycle. Furthermore, sirReal2 can suppress the deacetylation activity of SIRT2 and its downstream target PEPCK1, as well as mitochondrial metabolism and the RAS/ERK/JNK/MMP-9 pathway, limiting migration and invasion in GC cells. As a result, SirReal2 is expected to be a novel candidate for GC treatment 187.

**Isobavachalcone (IBC)** is the primary active ingredient in psoralen and possesses anticancer properties. IBC has been identified as a natural SIRT2 inhibitor, effectively decreasing SIRT2 enzyme activity by the formation of hydrogen bonds with VAL233 and ALA135. It is a promising natural lead chemical for the development of SIRT2-targeted inhibitors 278.

**Thiomyristoyl (TM)**, a thiomyristoyl lysine compound, is a SIRT2 inhibitor with excellent potency and specificity. Kinetics- and MS-based experiments demonstrated that TM is a mechanism-based inhibitor of SIRT2 capable of competing with substrates but not with NAD co-substrates. TM dose-dependently increased α-microtubulin acetylation without affecting p53 acetylation in breast cancer and CRC cell lines 263. SIRT2 inhibition promotes c-Myc ubiquitination and degradation. The anticancer effects of TM correlate with its ability to reduce c-Myc levels, and TM exhibits broad-spectrum anticancer activity in many cancer cell lines, with effects similar to SIRT2 knockdown 279.

**Kaempferol (KMP)** is a chemical flavonol derivative that is widely distributed in plants as a glycosidic ligand 280**.** KMP inhibits SIRT3 and SIRT6 in TNBC, thereby promoting breast cancer progression 281. SIRT3 maintains ROS levels to sustain proliferative and invasive phenotypes, thereby preventing apoptosis and promoting carcinogenesis in TNBC cells 282. SIRT6 regulates cancer metabolism by modulating both single- and double-stranded DNA break repair pathways and inflammation, promotes genomic stability and facilitates breast cancer progression 283.

**OSS_128167** is the first selective SIRT6 inhibitor identified by computerized screening 284. In DLBCL, selective OSS_128167-mediated SIRT6 blockade inhibited PI3K/Akt/mTOR signaling and produced similar anti-lymphoma effects compared to SIRT6 knockdown in DLBCL cells. Xenograft models treated with OSS_128167 showed inhibition of tumor growth. In addition, inhibition of SIRT6 increased the sensitivity of DLBCL cells to chemotherapeutic agents 285.

SIRT4 is located in mitochondria and is a potential therapeutic target for cancer and metabolic diseases, but effective and selective SIRT4 inhibitors have not been reported. The first potent and specific SIRT4 inhibitor was recently identified by exploiting SIRT4 de-HMGylation activity **compound 69**. The compound acts at low micromolar levels in cells and Selectivity for SIRT4 approximately 2-3 times that of SIRT1/2/3/5/6. SIRT4 plays a key role in suppressing tumorigenesis in a variety of cancer types (e.g. colorectal, breast, prostate), consistent with its low expression levels in these tumors. Therefore, the development of more effective SIRT4 inhibitors for cancer therapy could be a future approach in the realm of small molecule modulators targeting SIRT4 286.

The unique preference of SIRT5 for propionyl and succinyl groups in lysine residues in hydrolysed substrates has guided the design of specific SIRT5-regulated modulators. The H3K9TSu peptide, a thiosuccinimidyl peptide, became the first selective competitive inhibitor targeting SIRT5 287. Therefore, researchers screened and structurally optimised 5000 compounds to obtain the most potent and selective SIRT5 inhibitor reported to date, **compound 47**, which is to be further investigated 288. In addition to conventional SIRT5 inhibitors that compete with acyl lysine substrates, 3-thioureidopropionic acid derivatives significantly enhanced the thermal stability of SIRT5 and maintained isoform selection for SIRT5 289.

Nε-glutaryllysine-based compound **MC3482** inhibits SIRT5 and suppresses the desuccinylation activity of SIRT5 in human breast cancer cells (MDA-MB-231) and mouse myoblasts (C2C12) without affecting its expression. MC3482 exhibits dose-dependent inhibition of SIRT5-mediated desuccinylation in MDA-MB-231 cells and is selective for SIRT1 and SIRT3. In both cell lines tested, MC3482 at a concentration of 50 μM increased overall protein succinylation but not acetylation. MC3482 also resulted in enhanced succinylation, which led to the activation of glutaminase, thereby increasing cytosolic ammonia and glutamate levels, the latter of which triggers autophagy and mitochondrial autophagy 290.

In addition to the above SIRT5 inhibitors, there are several selective SIRT5 inhibitors (CG-220, CG-232) whose anti-tumor activity has not been further investigated. **CG-220** and **CG-232** address the poor solubility, low oral bioavailability, and ease of degradation of **Balsalazide**, an FDA-approved drug for the treatment of inflammatory bowel disease, by structurally modifying the drug, while enhancing its inhibitory activity against SIRT5. Unfortunately, only SIRT5 modulators have been reported for potential cancer treatment, and none have entered clinical trials 291.

**SIRT7 inhibitor 97491** up-regulates apoptosis via cysteine asparaginase-related proteins and inhibits cancer growth *in vivo*. It inhibits the deacetylase activity of SIRT7 and prevents tumor progression by increasing the stability of p53 through acetylation of the K373/382 site 292.

### Small-molecule modulators targeting SIRTs in clinical trials for cancer therapy

In light of a lack of subtype selectivity, modest potency, limited bioavailability, and poor pharmacokinetic (PK) and pharmacodynamic (PD) characteristics, many small molecule sirtuin inhibitors and activators are only in preclinical testing. The main small molecule modulators of SIRT1 have entered clinical trials. Among them, resveratrol and its derivatives have been the most intensively tested in clinical trials against cancer.

The first clinical trial to report resveratrol as a cancer treatment, a low-dose plant-derived resveratrol preparation or freeze-dried grape powder (GP) containing resveratrol inhibited the Wnt pathway *in vivo* in patients with colon cancer, and this effect was limited to the normal colonic mucosa 300. Patel and colleagues reported resveratrol and its metabolites in human tissues. HPLC/UV was used to identify and quantify the levels of resveratrol and its metabolites resveratrol-3'-O-glucuronide, resveratrol-4'-O-glucuronide, resveratrol-3'-O-sulfate, resveratrol-4'-O-sulfate, resveratrol glucuronide sulfate, resveratrol glucuronide sulfate, and resveratrol disulfate in colorectal resected tissue. In this study, 0.5 and 1.0 g dosages of resveratrol were found to significantly reduce tumor cell proliferation by 5% in all colon cancer patients 301. Zhu and colleagues investigated the impact of resveratrol on the methylation of breast cancer-related proteins in women who are at a higher risk of breast cancer. The effects of 5 or 50 mg trans-resveratrol twice daily (12 weeks) on the methylation of specific genes were compared to a placebo. The study found that with increased circulating levels of trans-resveratrol and resveratrol-glucuronide, as well as a decrease in prostaglandin E, methylation of RASSF-1a decreased2 (platinum group element 2) expression in the breast. Elevated levels of platinum group elements 2 and increased methylation of RASSF-1a are associated with disease progression from precancerous lesions to breast cancer. The varied criteria and longer follow-up of this trial may be advantageous in establishing whether short-term or continuous resveratrol supplementation is better for those with a greater likelihood of breast cancer 302.

In a phase 1 clinical trial of vorinostat combined with the sirtuin inhibitor nicotinamide in patients with relapsed or refractory lymphoma in diffuse large B-cell lymphoma, the overall remission rate was 24%. This was an unselected and heavily pretreated group of patients, most of whom received autologous SCT, allogeneic SCT, or both. In addition, 12 of 21 patients (57%) had stable aggressive disease 303. Combining nicotinamide with a first-generation EGFR-tyrosine kinase inhibitor (EGFR-TKI) in patients with stage IV lung cancer harboring an EGFR mutation could potentially improve patients' progression-free and overall survival, with significant survival benefits for female patients and those who have never smoked (NCT02416739) 304.

## Combination therapeutics with SIRT modulators

Combination therapy is a burgeoning approach in cancer treatment and has yielded remarkable outcomes. Combination therapeutics have the advantages of increased anticancer effects, reduced dose administration, and fewer adverse effects than single agents 305. For example, amurensin G, a potent inhibitor of SIRT1, results in the inhibition of FOXO1 and MDR1 protein levels in adriamycin-resistant breast cancer cells. Furthermore, amurensin G significantly increases cellular uptake of adriamycin and restores its ability to inhibit breast cancer cell-induced oncogenic growth under *in vitro* and *in vivo* conditions. Thus, amurensin G could be developed as a potential therapeutic agent, without significant toxicity, for MDR reversal in breast cancer in combination with other chemotherapeutic treatments 306. Likewise, SIRTs regulators may also show great potential in combination therapy of cancer **(Table 7)**.

### Mitigate drug resistance

The SIRT1 and SIRT2 inhibitors, **cambinol** and **EX-527**, were utilized in the context of hepatocellular cancer. Following therapy, increased levels of FOXO1 acetylation promoted the pro-apoptotic effect of tumor suppressor proteins and worsened p53's ability to induce apoptosis. Both medications increased apoptosis while decreasing colony size, cell migration, and viability. When used with traditional chemotherapeutic drugs, SIRT1 and SIRT2 inhibitors have the potential to overcome drug resistance during the treatment of HCC due to the down-regulation of P-gp and MRP3 in HepG2 cells 307.

### Improvement of efficacy

NSCLC is commonly treated with EGFR inhibitors (such as gefitinib or erlotinib) and platinum-based chemotherapy (such as cisplatin). The combination of cisplatin and erlotinib with the SIRT1 inhibitor** EX527** shown synergistic anticancer effects; however, in lung cancer, EX527-mediated inhibition of SIRT1 increased the efficacy of erlotinib by decreasing cell proliferation, augmenting DNA damage, and increasing apoptosis. Combination therapy may be a useful therapeutic approach for NSCLC, as evidenced by the combination therapy group's greater median survival time compared to the monotherapy or untreated groups 308.

The combination of AM with PAX at a fixed 1:1 ratio resulted in additive interactions in breast cancer cell viability. The two active compounds were employed independently to diminish cancer cell viability and proliferation while inducing apoptosis and cell cycle arrest. Another study discovered that cambinol increased sorafenib's inhibitory effect on HCC cell lines. Cell cultures treated with sorafenib with cambinol demonstrated greater declines in cell viability, proliferation, migration, invasion, apoptosis induction, and cell cycle arrest than cells treated with sorafenib individually. It has been shown that CAM-induced caspase-3/7, cyclin D1, and proliferative index-67 (Ki-67) protein production may be related with increased HCC cell sensitivity to sorafenib 309.

### Potential strategy

**SRT501** is a proprietary formulation of resveratrol with enhanced pharmacokinetic properties and higher oral bioavailability 176. Conversely, SRT2183 is a small molecule SIRT1 activator generated from non-natural products that differs structurally from resveratrol 177. In malignant cells, SRT501 and SRT2183 alone shown growth inhibitory and pro-apoptotic actions together with deacetylation of STAT3 and NF-κB and decreased levels of c-Myc protein. Although there was a higher suppression of c-Myc protein levels and H2A. X phosphorylation when the HDAC inhibitor pabinostat was combined with SRT2183, SRT501, or resveratrol, there was also an increase in the acetylation of H4 and p53. increased anticancer effects of SIRT1 activators in conjunction with pabinostat, i.e., enhanced antiproliferative and antisurvival effects produced by either drug alone 137.

**Paclitaxel (PAX)** is an anti-mitotic chemotherapeutic agent used in the treatment of different malignancies and is commonly used as a first-line chemotherapeutic agent in patients with breast cancer, especially the TNBC subtype. Limitations of PAX alone include low solubility and chemoresistance, as well as significant adverse effects such sensory neuropathy, histamine-mediated hypersensitivity reactions, and decreased drug delivery. On the other hand, in BC patients, the combination of the SIRT inhibitor AGK2 and PAX can essentially eradicate resistance to PAX and lower the dosage of PAX to minimize this medication's side effects. Additionally, glioma cell lines showed a cumulative antiproliferative effect from the combination of AGK2 and EX-527 310. The growth of A549 and H1299 cells was markedly reduced by the combination of sodium dichloroacetate (DCA) and AGK2. In pyruvate dehydrogenase α1 (PDHA1), AGK2 phosphorylated more lysine and dephosphorylated more serine, allowing AGK2 to work in concert with DCA. By switching from glycolysis to the mitochondrial OXPHOS, AGK2 caused metabolic remodeling. This included an increase in ROS production and oxygen consumption rate (OCR) and a decrease in lactate formation and glucose intake. Remodeling, which includes an increase in OCR and ROS creation along with a decrease in lactate synthesis and glucose consumption. The combination of AGK2 and DCA is known to enhance cancer inhibition when compared to monotherapy. This suggests a potential mechanism for the inhibitory effects of the two medications on NSCLC 311.

Conventional therapies utilize a large number of cytotoxic drugs such as cisplatin, doxorubicin, epirubicin, paclitaxel, and gemcitabine, which attack DNA replication and synthesis and preferentially target most large tumor cells. Unlike conventional chemotherapy, inhibition of SIRTs-mediated approaches along with conventional chemotherapy may contribute to enhanced mechanisms of cancer suppression. Among DNA-damaging agents, SIRT1 inhibitor (EX527) in combination with erlotinib has shown combined anti-lung cancer effects by reducing cancer cell proliferation and enhancing DNA damage. The combination of SIRT2 inhibitor (AGK2) and PAX eliminates PAX resistance, thereby minimizing the side effects of this drug. Thus, there is great potential for enhancement of the mechanisms of classical therapeutic agents currently used in cancer through a combination therapy approach. In addition, the new cancer machine learning algorithm REFLECT: Predicting optimal treatment combinations for cancer based on synergistic mutation profiling also provides new opportunities for compounds targeting SIRTs in combination therapies, potentially finding the best match for compounds targeting SIRTs.

## Future prospects and directions for targeting SIRTs in cancer therapy

SIRTs have a wide range of functional significance and are the targets of many drugs in clinical practice. SIRTs is closely related to programmed cell death, and intervening programmed cell death by regulating SIRTs may have unexpected effects. For instance, a growing body of research suggests that SIRTs can be involved in ferroptosis by affecting multiple aspects of redox homeostasis, iron metabolism, and lipid metabolism. Ferroptosis is an emerging non-apoptotic sort of regulated cell death that is substantially iron-dependent and characterized by cell membrane rupture 313. It was found that increased SIRT1 expression induced Nrf2-mediated antioxidant activity, which increased glutathione peroxidase 4 (GPX4) and glutathione (GSH) levels in HCC. In contrast, they mediate ferroptosis through the down-regulation of procalcitonin 20 (PCDH20) by decreasing the expression of the iron death-associated proteins GPX4 and GSH and by increasing intracellular iron levels and ROS 314. Likewise, SIRT6 promotes ferroptosis in pancreatic cells by upregulating ROS levels. In contrast, SIRT6 downregulation led to inactivation of the Keap1/Nrf2 signaling pathway and downregulation of GPX4, which resisted sorafenib-induced ferroptosis 315. Therefore, in tumor cells that are sensitive to ferroptosis, the therapeutic effect can be exerted by regulating SIRTs to induce ferroptosis. Similarly, SIRTs regulators can also exert synergistic potential in combination with ferroptosis inducers. The same holds true for other forms of programmed cell death, where the modulation of SIRTs or combinations thereof can potentiate the induction of programmed cell death for cancer therapy.

A growing body of evidence suggests that SIRTs play a crucial role in delaying cellular senescence and extending the lifespan of organisms by regulating a diverse range of cellular processes. Senescence is separated into replicative and premature forms, with the former caused by telomere wear and tear and the latter by depletion of critical cytokines such as oncogenes and tumor suppressors 316. SIRTs contribute to biological longevity by regulating several cellular functions. This includes preserving genomic integrity, improving DNA repair processes, and reducing age-related telomere erosion, which may play a role in cancer treatment. In the regulation of cell senescence and cellular DNA damage repair, appropriate regulation of SIRTs may disrupt the balance of tumor survival and provide a new way to resist tumor recurrence.

In the case of SIRTs with available structural data, the combination of different chemotypes from molecules with similar modes of action that have co-crystallized with the target protein, as well as scaffold hopping approaches, will most likely be critical in the future development of novel compounds with higher potency and selectivity. The amalgamation of structural data, computational approaches, and DNA-encoded libraries, as well as high-throughput tests, will enable faster assessment of vast chemical libraries, potentially leading to the development of novel isoform-selective chemotypes. Furthermore, the use of orthogonal biophysical techniques based on different detection methods (e.g., microscale thermophoresis, surface plasmon resonance, and native mass spectrometry) will likely allow for more accurate evaluation of the interactions and potency of new modulators while minimizing artifacts. The potential therapeutic success of SIRT-targeting modulators will require a deeper understanding of SIRT participation in certain disorders. Indeed, our understanding of SIRT biology remains restricted, and it can be difficult to reconcile the multiple - and sometimes contradictory - roles that multiple SIRTs in various diseases, including cancer. Likewise, viable candidate medications must be modified in terms of pharmacokinetics to achieve nanomolar potency and target selectivity. The combination of these characteristics is likely to produce new potential modulators that can be used not only as chemical probes for functional annotation of these amazing enzymes, but also as potential therapeutics for the treatment of an assortment of disorders, either alone or in combination with other drugs. In a nutshell, existing co-crystal structures, as well as cutting-edge approaches such as artificial intelligence-driven drug design and DNA-encoded libraries, have enormous potential for evaluating a more diverse chemical space for molecules with drug-like properties, which will facilitate the discovery of new SIRT modulators 317.

In order to improve targeting, the therapeutic efficacy of drugs is improved and clinical applications are facilitated by stabilizing compounds, overcoming barriers to cellular and tissue uptake, and increasing the biodistribution of drugs to their targets *in vivo*, while minimizing systemic toxicity 318. Tumor-targeted delivery systems, such as liposomes, nanoparticles, antibody-drug couplings (ADC), and exosomes, facilitate precise delivery of drugs to cancer cells, overcoming some of the current challenges associated with drug delivery, such as poor bioavailability, nonspecific toxicity, and rapid clearance from the body 319. Moreover, given the functional similarities and differences between different SIRTs, developing small molecules that target multiple SIRTs may also have better benefits in specific situations than targeting a single SIRT. For example, Allotinib can effectively inhibit VEGFR, PDGFR, FGFR, c-Kit and other signaling pathways, so it has multiple effects of anti-tumor angiogenesis and inhibition of tumor growth, showing good clinical effects in the treatment of NSCLC. Therefore, in the development of drugs targeting SIRTs, they must be tailored based on tumor characteristics. It is not a matter of higher targeting being better or having more targets being superior. Similar to chess, victory can only be achieved by understanding both oneself and the enemy.

## Conclusions

The SIRT family, comprising seven isoforms in mammals, plays a crucial role as important deacetylases with significant epigenetic functions attributed primarily to their deacetylase activity. Each isoform exhibits distinct subcellular localization and biological functions. Given their pivotal involvement in various biological processes such as cellular differentiation, transcriptional regulation, cell cycle progression, apoptosis, inflammation, metabolism, neurological and cardiovascular physiology, as well as cancer development and progression; SIRTs have garnered increasing attention.

The epigenetic and non-epigenetic roles of SIRTs in cancer are increasingly receiving more attention. Various studies have shown that different SIRTs exhibit different expression patterns according to pathological subtype, tumor grade and stage. It can be concluded that SIRTs can be used as biomarkers for cancer patients. To understand the intricate role that SIRT plays in various malignancies and cell types, as well as the circumstances in which particular SIRTs act as tumor promoters or repressors, more research is required. Indeed, most studies have focused primarily on the development of SIRT1 and SIRT2 regulators, probably because of the large amount of structural, biochemical, and biophysical data that exist for these isoenzymes. However, there is currently a lack of effective inhibitors targeting SIRT3-7. Moreover, the number of activators for SIRTs is relatively limited compared to the abundance of inhibitors. But in general, activators have better therapeutic potential than inhibitors. For this reason, scientific approaches to drug design are especially crucial. In addition, due to the limitations of clinical trials and drug development, many modulators of SIRTs are still in the experimental pharmacology stage and have not yet entered clinical practice or faced the challenge of translating from *in vitro* to *in vivo* experiments. Clearly, the safety, dose modulation, and human toxicity of these modulators require further improvement. Future research should focus on increasing the selectivity and bioavailability of novel SIRT modulators in order to precisely regulate SIRT activity while reducing negative effects. This can be accomplished by improving the drug's molecular structure or inventing novel delivery mechanisms. As a result, more study into SIRT activators and inhibitors is required, which will eventually reveal the entire therapeutic potential of SIRTs molecules.

As for the application of SIRTs targeted drugs in cancer therapy, although there are few clinical studies at present, they still have very great potential. A single spark can start a prairie fire, as long as you find a good point, it will certainly open brilliant flowers. SIRTs regulate a variety of cell metabolites, such as ROS, lactic acid, etc., and participate in the shaping of the genome as well as the cell microenvironment, all of which are crucial for cancer treatment. For example, in chemotherapy, the most important problems at present are drug resistance and the toxic side effects caused by chemotherapy. Chemotherapy generally works through DNA damage, or by inducing programmed cell death, which also provides opportunities for the use of SIRTs regulators. For example, long-term treatment with paclitaxel may reduce its apoptosis-inducing ability, and long-term use of platinum-based chemotherapy drugs may also reduce its ability to damage DNA. Chemotherapy can also cause severe nerve damage. Considering the differences in metabolic patterns between normal cells and cancer cells, at this time, if the activator of SIRT3 is combined, the anti-tumor effect of paclitaxel and platinum can be enhanced not only by activating programmed cell death (apoptosis, autophagy associated cell death), but also by enhancing the mitochondrial function of nerve cells to reduce the neurotoxicity of chemotherapy drugs. In radiotherapy, the combined use of SIRTs regulators can inhibit DNA damage repair, aggravate DNA damage, and achieve the purpose of synergism. In immunotherapy, the effectiveness of treatment depends mainly on the ability of an individual's immune system. Currently, immunotherapy, especially CAR-T, is mostly effective in hematoma. At this time, SIRTs activators can also reshape the immune microenvironment by regulating mitochondrial metabolism and enhance the effect of hemoma immunotherapy. As for immune checkpoint inhibitors, mainly PD1/PD-L1 monoclonal antibodies can be used for the treatment of a variety of cancers, including solid tumors. Immune escape is a major difficulty in immunotherapy. However, based on the metabolic reprogramming characteristics of tumor cells, SIRTs activation can not only directly kill sensitive cancer cells, but also identify and kill escaped tumor cells.

**Figure 7:**
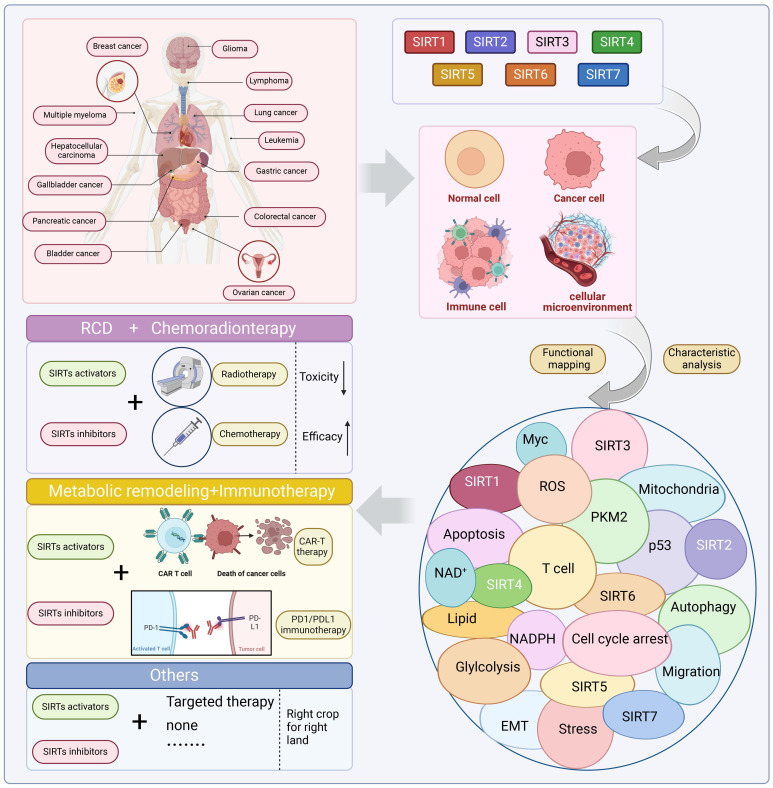
The conceptualization and prospective avenues for targeting SIRTs in cancer therapy.

In a nutshell, SIRTs can be used as promising target molecules as potential biomarkers for the diagnosis and prognosis of cancer patients. SIRTs as epigenetic drugs have shown potential as stand-alone treatments for cancer and have also been found to work well in combination with other therapies (chemotherapy, radiotherapy and immunotherapy) to help overcome treatment resistance **(Figure 7)**. Looking at the big picture from God's perspective, finding the most appropriate way to regulate SIRTs, whether it is a small adjustment or a drastic change, will open up new avenues for cancer treatment.

## Figures and Tables

**Table 1:** Classification, function and characteristics of mammal SIRTs

SIRT	Class	Cellular localization	Enzymatic activity	Histone deacetylation target	Biological function	Refs.
SIRT1	I	Nucleus and cytoplasm	a. Deacetylase b. Depropionylase	H1-K26Ac H3-K9Ac H4-K16Ac	a. Chromatin modification b. DNA repair c. Cell cycle regulation d. Cell metabolism and survival	32
SIRT2	I	Nucleus and cytoplasm	a. Deacetylase b. Demyristoylase c. Depropionylase	H3-K18Ac H3-K56Ac H4-K16Ac	a. Cell cycle regulation b. Microtubule dynamics c. Inflammation d. Differentiation	33
SIRT3	I	Mitochondria	a. Deacetylase b. Depropionylase	H3-K56Ac H4-K14Ac	a. Apoptosis b. Nuclear gene expression c. Control of metabolism	2
SIRT4	II	Mitochondria	a. Deacetylase b. ADP ribosyltransferase c. Biotinidase d. Lipoamidase	H4-K16Ac	a. Resistance b. Genomic stability c. Energy metabolism	34,35
SIRT5	III	Mitochondria	a. Deacetylase b. Demethylase c. Desuccinylase d. Glutaminase	Unknown	a. Mitochondrial metabolism b. Amino acid degradation c. Cellular respiration d. Reactive oxygen species management	36
SIRT6	IV	Nucleus	a. Deacetylase b. ADP-ribosylation c. Defattyacylation	H3-K9Ac H3-K18Ac H3-K56Ac	a. Cell proliferation b. Energy metabolism c. DNA damage repair d. Stem Cell Differentiation	37
SIRT7	IV	Nucleolus	a. Deacetylase b. Desuccinylase	H3-K18Ac	a. DNA Repair b. RNA transcription c. Metabolism regulation	38

**Table 2:** The regulation of SIRTs

SIRT	Classification	Regulator	Regulatory Mechanism	Refs
**SIRT1**	Transcription factor	FOXO3a	FOXO3a binds to p53 in the SIRT1 promoter	39
	Transcription factor	C-Myc	C-Myc binds to the SIRT1 promoter and induces SIRT1 transcription	56
	Transcription factor	NCAPD3	NCAPD3 targets the promoter of SIRT1 through TFII I anchoring	40
	Transcription factor	HIC1	HIC1 inhibits SIRT1 expression and regulates the DNA damage response	57
	Endogenous product	AROS	AROS interacts with and activates SIRT1	58
	MicroRNA	MiR-22	MiR-22 inhibits SIRT1 translation and enhances the efficacy of arabinogalactan treatment in acute myeloid leukemia cells	59
	MicroRNA	MiR-34a	MiR-34a targets SIRT1, increases levels of acetylated p53 and inhibits cell migration and invasion	60
	MicroRNA	MiR-122	MiR-122 inhibits SIRT1 expression by binding to the 3′-UTR of SIRT1	61
	MicroRNA	MiR-200a	MiR-200a targets SIRT1 3'-UTR to reduce its expression and inhibit mesenchymal transition (EMT)	62
	MicroRNA	MiR-181	MiR-181c and -181d target SIRT1 to suppress chemotherapy resistance	63
	MicroRNA	MiR-449a	MiR-449a inhibits SIRT1 expression, enhances p53 acetylation, and promotes apoptosis	42
	MicroRNA	MiR-30a-5p	MiR-30a-5p targets SIRT1 and activates the NF-κB/NLRP3 signaling pathway, leading to apoptosis	64
	Long non-coding RNA	PRLB	PRLB inhibits miR-4766-5p expression, thereby negatively regulating SIRT3	65
	Long non-coding RNA	DACT3-AS1	DACT3-AS1 targets SIRT1 to inhibit cell proliferation, migration and invasion	66
	Circular RNA	circ0088036	circ0088036 serves as a sponge of miR-140-3p and upregulates the expression of SIRT1	67
	Circular RNA	circ0001946	circ0001946 promotes lung adenocarcinoma progression by upregulating SIRT1 and activating the Wnt/β-catenin signaling pathway via sponge miR-135a-5p	48
	Protein	ClpP	ClpP activates SIRT1 to regulate vascular smooth muscle cells	52
	Protein	Zyxin	The interaction of zyxin and SIRT1 enhances its expression and inhibits EMT	68
	Protein	SMURF2	Ubiquitination-mediated degradation of SIRT1 by SMURF2 suppresses CRC cell proliferation and tumorigenesis	69
	Protein	SENP1	SENP1 sumoylates SIRT1 and promotes its expression	70
**SIRT2**	Transcription factor	Nkx2.2	Nkx2.2 passages bind to the SIRT2 promoter and negatively regulate its expression	71
	Transcription factor	HIF1α	HIF1α interacts with HRE in the SIRT2 promoter to repress its expression	41
	MicroRNA	MiR-200c-5p	MiR-200c-5p targets SIRT2 and downregulates its expression	43
	MicroRNA	MiR-140-5p	MiR-140-5p targets SIRT2 to promote myocardial oxidative stress	72
	MicroRNA	MiR-212-5p	MiR-212-5p targets SIRT2 3'-UTR, down-regulates SIRT2, and promotes colorectal cancer progression	73
	MicroRNA	MiR-221-3p	MiR-221-3p targets SIRT2 3'-UTR, down-regulates SIRT2	74
	MicroRNA	MiR-200c-5p	MiR-200c-5p targets SIRT2 and downregulates its expression	43
	MicroRNA	MiR-140-5p	MiR-140-5p directly targets SIRT2 downregulates its expression	72
	Long non-coding RNA	CASC2	CASC2 inhibits miR-18a expression and down-regulates SIRT2/ROS pathway	75
	Long non-coding RNA	NEAT1	NEAT1 inhibits miR-221-3p and causes SIRT2 activation	74
	Protein	C-Src	C-Src interactes with SIRT2 and phosphorylates SIRT2, reducing the protein level and stability of SIRT2.	41
	Protein	CDK	CDK phosphorylates SIRT2 at the Ser331 site and inhibits catalytic activity	41
	Protein	CBL	CBL increases protein levels and stability of SIRT2 through ubiquitination	41
**SIRT3**	Transcription factor	NF-κB	NF-κB binds to the SIRT3 promoter to enhance its expression	76
	Transcription factor	ZMAT1	ZMAT1 binds to the promoter of SIRT3 and activates transcription	77
	Transcription factor	ZEB1	ZEB1 inhibits SIRT3 promoter activity	76
	Transcription factor	SNAI1	SNAI1 inhibits SIRT3 promoter activity	76
	Endogenous product	4-Hydroxynonenal	4-Hydroxynonenal inhibits SIRT3 activity by occupy its zinc-binding residue Cys 280	78
	SUMOspecific protease	SENP1	SENP1 de-SUMOylates and activates SIRT3	79
	MicroRNA	MiR-23b-3p	MiR-23b-3p targets the 3'UTR of SIRT3 and inhibits SIRT3 expression	80
	MicroRNA	MiR-494-3p	MiR-494-3p targets the 3'UTR of SIRT3 and inhibits SIRT3 expression	80
	MicroRNA	MiR-31	MiR-31 directly targets SIRT3 to repress its expression	81
	MicroRNA	MiR-224	MiR-224 targets the 3'UTR of SIRT3 and decreases SIRT3 protein level	82
	MicroRNA	MiR-421	MiR-421 promotes PC progression by regulating the SIRT3/H3K9Ac/HIF-1α axis	83
	MicroRNA	MiR-708-5p	MiR-708-5p inhibits the progression of pancreatic ductal adenocarcinoma by targeting Sirt3	84
	MicroRNA	MicroRNA-761	MicroRNA-761 targets SIRT3 and enhances pazopanib resistance in synovial sarcoma	85
	Long non-coding RNA	TUG1	TUG1 sponges miR-145 to promote cancer progression via Sirt3/GDH axis	86
	Protein	TRAP1	Interaction between TRAP1 and Sirtuin-3 Regulates Mitochondrial Respiration and Oxidative Stress Recording	87
**SIRT4**	MicroRNA	MiR-503	MiR-503 targets SIRT4 and inhibits SIRT4 expression	88
	MicroRNA	MiR-15a-5p	MiR-15a-5p enhances the malignant phenotypes of colorectal cancer cells through the STAT3/TWIST1 and PTEN/AKT signaling pathways by targeting SIRT4	44
	MicroRNA	MiR-424	MiR-424 targets SIRT4 and inhibits SIRT4 expression in the regulation of CDDP sensitivity of bladder cancer cells	89
	MicroRNA	MiR-503	MiR-503 targets SIRT4 and inhibits SIRT4 expression	88
	MicroRNA	MiR-15a-5p	MiR-15a-5p enhances the malignant phenotypes of colorectal cancer cells through the STAT3/TWIST1 and PTEN/AKT signaling pathways by targeting SIRT4	44
	MicroRNA	MALAT1	MALAT1 regulates miR-93-5p to modulate SIRT4	90
	Circular RNA	OMA1	OMA1 promotes BC progression by sponging miR-1276 and upregulating SIRT4 expression	49
	Protein	OPA1	OPA1 interacts with SIRT4 to negatively regulate mitochondrial autophagy	91
	Protein	PAK6	PAK6 promotes ubiquitination degradation of SIRT4 and affects apoptosis in prostate cancer cells	92
	Protein	CtBP	CtBP targets SIRT4 and inhibits SIRT4 expression to maintain cancer cell growth and metabolic homeostasis	53
**SIRT5**	Transcription factor	ATF4	ATF4 binds to SIRT3 to enhance its expression	93
	Transcription factor	KLF4	KLF4 binds to the SIRT4 promoter and promotes its transcription	94
	Transcription factor	UHRF1	Inhibition of SIRT4 expression by UHRF1 promotes aerobic glycolysis and proliferation of PC cells	95
	MicroRNA	MiR-455	MiR-455 targets SIRT5 and inhibits SIRT5 expression	96
	MicroRNA	MiR-27b-5p	MiR-27b-5p targets SIRT5 and inhibits SIRT5 expression	46
	MicroRNA	MiR-299-3p	MiR-299-3p functions as a tumor suppressor via targeting SIRT5 in HCC	97
	MicroRNA	MiR-3677-3p	MiR-3677-3p targets SIRT5 and inhibits SIRT5 expression	98
	Long non-coding RNA	LINC01234	LINC01234 Adsorption of miR-27b-5p targets SIRT5 and affects OCSCs differentiation and self-renewal	46
	Long non-coding RNA	SNHG14	LncRNA SNHG14 aggravates invasion and migration as ceRNA via regulating miR-656-3p/SIRT5 pathway in HCC	99
	Circular RNA	ATL1	Circ-ATL1 silencing reverses the activation effects of SIRT5 on smooth muscle cellular proliferation, migration and contractility in intracranial aneurysm by adsorbing miR-455	96
	Circular RNA	CircLRWD1	CircLRWD1 regulates SIRT5 expression through adsorption of miR-507	45
	Protein	SLC25A51	SLC25A51 promotes HCC progression by driving aerobic glycolysis through activation of SIRT5	54
**SIRT6**	Transcription factor	FOXA2	Coordination of FOXA2 and SIRT6 suppresses the HCC progression through ZEB2 inhibition	100
	Transcription factor	FOXO3a	FOXO3a binds to the SIRT6 promoter to enhance its expression	101
	MicroRNA	MiR-25	miR-25 targets SIRT6 and inhibits its expression	102
	MicroRNA	MiR-33a	Suppression of SIRT6 by MiR-33a facilitates tumor growth of glioma through apoptosis and oxidative stress resistance	103
	MicroRNA	MiR-122	MiR-122 binds to 3′-UTR to inhibit the expression of SIRT6	61
	MicroRNA	MiR-34a-5p	MiR-34a-5p suppresses cutaneous squamous cell carcinoma progression by targeting SIRT6	104
	MicroRNA	MiR-654-5p	MiR-654-5p regulates cell progression and tumor growth through targeting SIRT6 in osteosarcoma	105
	Long noncoding RNA	HIF1A-AS2	HIF1A-AS2 promotes cell survival and migration by regulating SIRT6 expression in osteosarcoma via sponge miR-33b-5p	47
	Long noncoding RNA	HOXC-AS3	Binding of HOXC-AS3 to SIRT6 prevents contact inhibition of HIF1α, leading to reprogramming of metabolic pathways	106
	Long noncoding RNA	HMMR-AS1	HMMR-AS1 promotes proliferation and metastasis of lung adenocarcinoma by regulating MiR-138/sirt6 axis	107
	Circular RNA	CircITCH	CircITCH sponges miR-330-5p to ameliorate doxorubicin-induced cardiotoxicity through upregulating SIRT6	50
**SIRT7**	Protein	PYCR1	PYCR1-catalyzed NAD production stimulates SIRT7 deacetylation activity on H3K18ac, thereby inhibiting gene transcription	108
	Protein	CYP2E1/ ROS	CYP2E1/ ROS activates SIRT7 transcription	109
	MicroRNA	MiR-21-5p	MiR-21-5p promotes sorafenib resistance and HCC progression by regulating SIRT7 ubiquitination	110
	MicroRNA	MiR-148b	MiR-148b binds to 3′-UTR to inhibit the expression of SIRT7	111
	MicroRNA	MiR-770	MiR-770 binds to 3′-UTR to inhibit the expression of SIRT7	55
	MicroRNA	MiR-125a-5p	MiR-125a-5p binds to 3′-UTR to inhibit the expression of SIRT7	112
	MicroRNA	MiR-125b	MiR-125b binds to 3′-UTR to inhibit the expression of SIRT7	112
	Long noncoding RNA	AC007207.2	AC007207.2 promotes OS progression through miR-1306-5p sponge upregulation of SIRT7 expression	55
	Long noncoding RNA	LINC00886	LINC00886 sponges upregulation of SIRT7 expression	113
	Long noncoding RNA	AC007207.2	AC007207.2 promotes malignant properties of osteosarcoma via the miR-1306-5p/SIRT7 Axis	114
	Circular RNA	CircPVT1	Downregulation of circular RNA circPVT1 restricts cell growth of HCC through downregulation of Sirtuin 7 via microRNA-3666	115
	Protein	DDX3X	Interaction between SIRT7 and X-linked DEAD-box helicase 3 (DDX3X) promotes PD-L1 expression and thus tumor growth	55
	Protein	AKT/ p70S6K1	Interaction between SIRT1 and AKT or p70S6K1 for tumor progression	55
	Protein	GRIM-19	SIRT7 triggers PCAF-mediated ubiquitination of MDM2 and ultimately stabilizes p53 protein by interacting with GRIM-19	55

**Table 3:** Tumor suppressor role of SIRTs

SIRT	Cancer type	Regulator	Mechanism of action	Refs
SIRT1	Cervical cancer	APE1	Regulates cellular base excision repair	116
	Skin cancer	XPA	Deacetylates XPA, enhancing NER activity	117
	Skin cancer	XPC	AKT-dependent nuclear localization of inhibitory proteins	118
	Lymphoma	PP4	Regulates DNA damage signaling	119
	Chronic myeloid leukemia	WRN	Promotes HR	120
	Colorectal cancer	SAMHD1	Promotes DNA terminal excision and HR	121
	Chronic myeloid leukemia	KU70	Promotes NHEJ	122
	Colon cancer and melanoma	HINT1	Promotes the tumor suppressor activity of HINT1	123
	Glioma	NOX2/NAT10	Promotes DPT-induced parthanatos	124
	Pancreatic cancer	WEE1	Sensitizes cancer cells to WEE1 inhibition	127
	Breast cancer	Smad4	Inhibits EMT	126
	Glioma	ATF3	Sensitizes to Ferroptosis	125
SIRT2	NSCLC	SKP2	Increases expression of cell cycle protein-dependent kinase inhibitors	129
	Breast Cancer	Prdx-1	Promotes ROS level	131
	CRC	IDH1	Regulates SRC transcription	133
	HCC and breast cancer	CDH1/CDC20	Maintains mitosis and genome integrity	132
	Colorectal cancer	FOXO1/Atg7	Promotes autophagic processes	135
SIRT3	Breast Cancer	PHD	Controls the expression of glycolytic genes	136
	Breast cancer and leukemia	PDC	Inhibits glycolysis pathway	137
	Pancreatic	GOT2	Controls the glycolytic pathway	139
	Breast Cancer	CYPD	Inhibits glycolysis pathway	172
	Breast Cancer	p53	Inhibits glycolysis in wt-p53 cancer cells	138
	Hepatocellular cancer	GSK-3β	Promotes apoptosis	140
	HCC	Bax	Induces Bax expression and mitochondrial translocation	140
	Breast and prostate cancer	Skp2	Leads to enhanced cell proliferation, migration and tumorigenesis	141
	Osteosarcoma and cervical cancer	Histone H3 lysine 56	Induces non-homologous end joining	142
	Colorectal cancer	LONP1	Reduces the energy supply of OXPHOS	143
SIRT4	B Cell Lymphoma	GDH	Inhibits glutamate metabolism	148
	Colorectal and prostate cancer	mTORC1	Destabilization of CREB2 inhibits SIRT4 protein levels	150
	Breast Cancer	MTHFD2	Inhibits ROS level	152
	NSCLC	Drp1	Alters mitochondrial morphology	153
	Gastric cancer	p-ERK, cyclin D and cyclin E	Induces G1 cell cycle arrest	149
	Pancreatic ductal adenocarcinoma	p53	Induces autophagy	154
SIRT5	PDAC	GOT1	Attenuates glutamine and glutathione metabolic pathways	155
	Gastric cancer	OGDH	Increases ROS levels and NADP/NADPH ratio	156
	Gastric cancer	CDK2	Inhibits the positive regulation of aerobic glycolysis	157
	Lung cancer	STAT3	Inhibits pyruvate metabolism	158
	Colonic tumor	P53	Glycolytic reprogramming	159
SIRT6	HCC	PKM2	Regulates glycolysis	163
	PDAC	Lin28b	Inhibits downstream tumor proteins	164
	Breast cancer	Twist1	Inhibits EMT and metastasis	173
	NSCLC	FOXA2	Inhibits proliferation	100
	HCC	ERK1/2	Suppresses cancer cell growth	165
SIRT7	Breast Cancer	TGF-β	Suppresses EMT	170
	Bladder cancer	CHD1	Suppresses EMT	166
	OSCC	SMAD4	Suppresses EMT	168
	Breast Cancer	EST1	Suppresses EMT	167
	Breast Cancer	LAP2α	Induces chromosomal instability	171

**Table 4:** Oncogenic role of SIRTs

SIRT	Cancer type	Substrate	Mechanism of action	Refs
SIRT1	Pancreatic cancer	CRL4B	Represses the promoters of FOXO3 and GRHL3	176
	Melanoma	DNMT3B	Promotes the silencing of Mxd1	177
	Pancreatic cancer	Ptfla/β-catenin	Regulates ADM	175
	Melanoma	Snail/Twist	Promotes autophagy	177
	Melanoma	Beclin 1	Promotes autophagic degradation of E-cadherin	177
	Acute myeloid leukemia	NOTCH1	Mediates resistance to NOTCH1 inhibition	181
	T-ALL	CDK2	Promotes cell cycle	180
	CRC	MCU	Causes mitochondrial Ca^2+^ overload and depolarization and promotes apoptosis.	181
	Colorectal cancer	MiR-1185-1	Increases stemness and invasiveness	180
SIRT2	Breast Cancer	p53	Affects the regulation of G1/S and G2/M	183
	Breast Cancer	ALDH1A1	Promotes the growth of stem cells	184
	Breast Cancer	PKM2	Regulates glycolytic metabolism	185
	NSCLC	PGAM	Enhances NADPH production	186
	Gastric cancer	PEPCK1	Activates RAS/ERK/JNK/MMP-9 pathway	187
	HCC	β-catenin	Targets the Akt/GSKa/β-catenin pathway	188
SIRT3	B Cell Malignancies	IDH2	Regulates ROS levels	189
	Cervical cancer	ACC1	Promotes lipid metabolism.	190
	NSCLC	P53	Promotes p53 degradation	191
	Colorectal cancer	SHMT2	Inhibits lysosomal degradation	192
	Breast cancer	PYCR1	Involves in amino acid metabolism	193
	Neuroglioma	GLDC	Promotes glycine catabolism	194
	Chronic lymphocytic leukemia	MnSOD	Eliminates ROS	195
	DLBCL	GDH	Induces autophagy and cell death	198
	Non-small cell carcinoma	RIF1	Facilitates transfers and EMT	197
SIRT4	HCC	GNPAT	Promotes lipid metabolism	200
	Breast Cancer	STAT3	Inhibits phosphorylation and nuclear translocation of Y705 in STAT3	201
	Prostate cancer	ANT2	PAK6-SIRT4-ANT2 complex inhibits apoptosis	92
SIRT5	Colorectal cancer	TKT	Enhances DNA damage.	202
	Breast Cancer	GLS	Increases carbon and nitrogen levels	203
	Colorectal cancer	GLUD1	Enhances catabolism of glutamine	204
	HCC	E2F1	Promotes proliferation and invasion	16
	Gastric cancer	2-oxoglutamate dehydrogenase	Inhibit the growth and migration	16
	Gastric cancer	S100A10	Promoting gastric cancer invasion and migration	16
	HCC	Cytochrome C	Inhibits cell apoptosis	206
	Colorectal cancer	LDHB	Promotes autophagy and tumorigenesis	16
	Colorectal cancer	SHMT2	Catalyzes the catabolism of serine and provides methyl for cellular methylation reactions	205
SIRT6	HCC	Bax	Induces H3K9 deacetylation to block Bax transcription	207
	Multiple myeloma	H3K9	Regulates MAPK genes conferring effect on DNA damage resistance	209
	colorectal cancer	NDRG1	Inhibits apoptosis	210
	NSCLC	HIF-1α/HK2	Increases glycolysis	211
	HCC	PKM2	Deacetylates PKM2 to inhibit oncogenic function	163
SIRT7	Breast Cancer	ELK4	Acts on H3K18ac deacetylation in promoters	38
	HCC	USP39	USP39 Deacetylation	212
	Lung cancer	p53	Binds to the NOXA promoter and reduces its transcription	218
	HCC	OAT2	Reduces H3K18ac levels and decreases 5-FU uptake	17
	Breast Cancer	p38	Activates the MAPK pathway	167
	Ovarian cancer	GATA4	Activates the Wnt signaling pathway	182
	NSCLC	ARF	Promoting the proteasome-dependent degradation of tumor suppresser	215
	Pancreatic cancer	GLUT3	Interacts with the enhancer region of GLUT3 and desuccinylates H3K122	217
	HCC	MST1	Represses transcription and triggers acetylation-dependent ubiquitination and protein degradation	214

**Table 5:** Small molecule activators of SIRTs

Compound	Chemical Structure	Mechanism	Target	Cancer type	Activity	Refs
Resveratrol	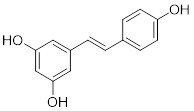	Resveratrol increases SIRT1, SIRT3, SIRT5, SIRT7 expression and activity	SIRT1, SIRT3, SIRT5, SIRT7	Colorectal cancer, breast cancer	EC_1.5_(SIRT1): 46.2 μM EC_1.5_(SIRT3): >300 μM	247
Quercetin	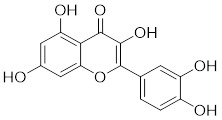	Quercetin increases SIRT1 expression	SIRT1, SIRT5, SIRT6	Lung cancer, NSCLC	EC_50_: 1.2 mM	248
SRT2183	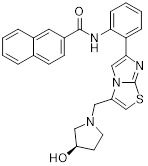	SRT2183 increases SIRT1 expression	SIRT1	Malignant lymphoma	EC_1.5_: 0.36 μM	249
SRT1720	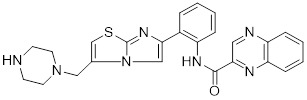	SRT1720 activates SIRT1-AMPK signaling	SIRT1	Pancreatic Cancer, multiple myeloma	EC_1.5_: 0.16 μM	233
SRT1460	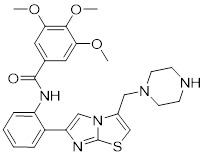	SRT1460 activates SIRT1-AMPK signaling	SIRT1	Pancreatic Cancer	EC_1.5_: 2.9 μM	237
SRT3025	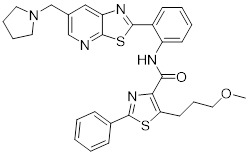	SRT3025 increases SIRT1 expression	SIRT1	Pancreatic Cancer	EC_1.5_: <1 μM	237
SCIC2.1	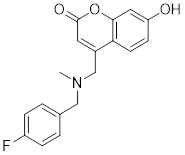	SCIC2.1 regulates SIRT3 to reduce the expression of SOD2	SIRT1, SIRT3	Liver cancer	Unknown	218,238
ADTL-SA1215	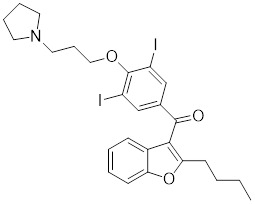	ADTL-SA1215 increases SIRT3 expression	SIRT3	Triple Negative Breast Cancer	Unknown	239
MC3138	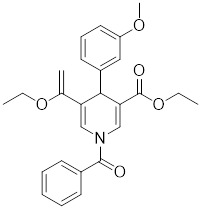	MC3138 increases SIRT3 expression	SIRT5	Pancreatic Cancer	EC_1.5_: ~1.5x SIRT5 active at 10 μM	155
Cyanidin	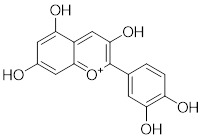	Cyanidin increases SIRT6 expression	SIRT6	Colon, skin and lung cancers	Unknown	250
UBCS039	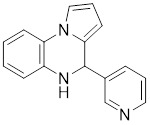	UBCS039 binds to the SIRT6-specific acyl channel pocket	SIRT6	NSCLC, colon cancer, epithelial cervical cancer	EC_50_: 38 μM	245,244
MDL-811	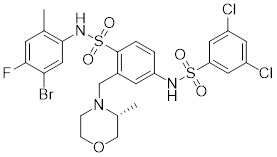	MDL-811 activates SIRT6 deacetylation	SIRT6	Colorectal cancer	EC_50_: 7.1 μM	221
MDL-800	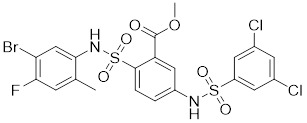	MDL-800 activates SIRT6 deacetylation	SIRT6	NSCLC	EC_50_: 10.3 μM	246

**Table 6:** Small molecule Inhibitors of SIRTs

Compound	Chemical Structure	Mechanism	Target	Cancer type	IC_50_	Refs
Nicotinamide	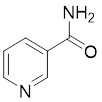	Competitive SIRTs Inhibitors	SIRT1, SIRT2	Meloanoma	SIRT1: 50-180 μM SIRT2: 2 μM	254,252
Sirtinol	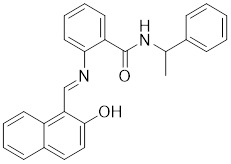	Competitive SIRTs Inhibitors	SIRT1, SIRT2	Breast cancer	SIRT1: 131 μM SIRT2: 57.7 μM	257,255
Salermide	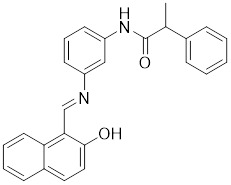	Competitive SIRTs Inhibitors	SIRT1, SIRT2	Colorectal cancer, glioblastoma multiforme	SIRT1 :42.8 μM SIRT2: 25 μM	260
Cambinol	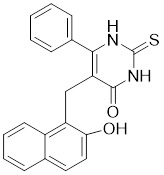	Competitive SIRTs Inhibitors	SIRT1, SIRT2	Lymphomas	SIRT1: 56 μM SIRT2: 59 μM	266,293
Selisistat	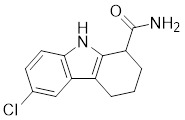	Competitive SIRTs Inhibitors	SIRT1	Breast cancer, ovarian cancer, cervical cancer	123 nM	262
AGK2	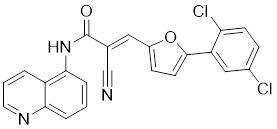	Chemical Library Screening-based SIRTs Inhibitors	SIRT1, SIRT2, SIRT3	HCC	SIRT1: 30 μM SIRT2: 3.5 μM SIRT5: 91 μM	294
Tenovin-1	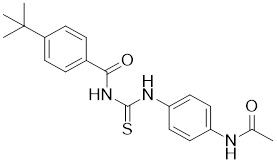	Chemical Library Screening-based SIRTs Inhibitors	SIRT1, SIRT2	Ewing's sarcoma	Unknow	269,295
SPC-180002	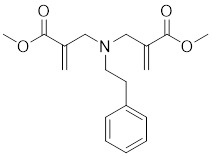	Impairing mitochondrial function and redox homeostasis	SIRT1, SIRT3	Unknown	SIRT1: 1.13 μM SIRT3: 5.41 μM	271
Tenovin-6	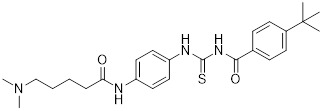	Chemical Library Screening-based SIRTs Inhibitors	SIRT1, SIRT2, SIRT3	Uveal melanoma, Diffuse large B-cell lymphoma	SIRT1: 21 μM SIRT2: 10 μM SIRT3: 67 μM	296,297
Inauhzin	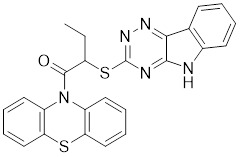	Structure-based SIRTs Inhibitors	SIRT1	Lung and colon cancer	0.7-2 μM	276,298
SirReal2	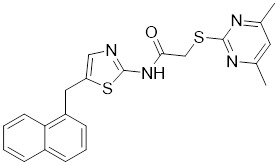	Structure-based SIRTs Inhibitors	SIRT2	Lung, colorectal, breast, lymphoma and cervical cancer	0.14-0.44 μM	299,253
Isobavachalcone (IBC)	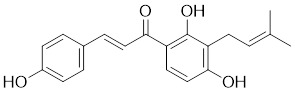	Natural lead compound	SIRT2	TNBC	0.84 μM	233
Kaempferol	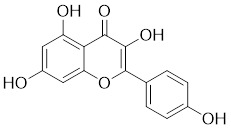	Other	SIRT3, SIRT6	Breast cancer	Unknow	281
Thiomyristoyl	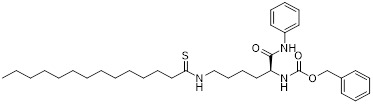	Other	SIRT1, SIRT2	Breast cancer	SIRT1: 98 μM SIRT2: 28 nM	279
OSS_128167	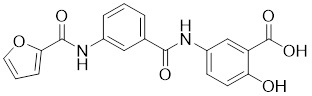	Other	SIRT1, SIRT2, SIRT6	Multiple myeloma	SIRT1: 1578 μM SIRT2: 751 μM SIRT6: 89 μM	284
						
Compound 69	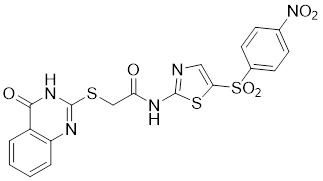	Competitive SIRTs Inhibitors	SIRT4	Unknown	16 μM	286
H3K9TSu peptide	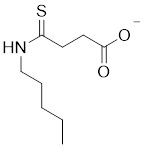	Competitive SIRTs Inhibitors	SIRT5	Unknown	5 μM	287
Compound 47	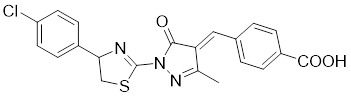	Competitive SIRTs Inhibitors	SIRT5	Unknown	0.21 μM	288
MC3482	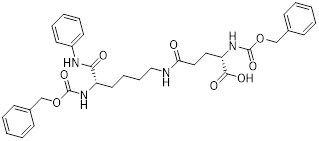	Other	SIRT5	Breast cancer	Unknow	290
Balsalazide	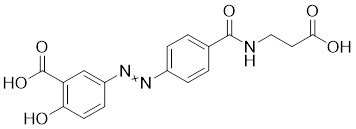	Other	SIRT5	Unknown	5.3 μM	291
CG-220	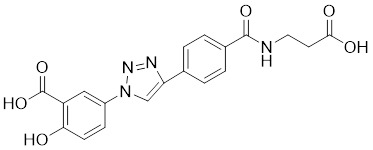	Other	SIRT5	Unknown	7.4 μM	291
CG-232	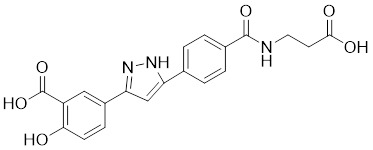	Other	SIRT5	Unknown	SIRT5: 7.7 μM	291
SIRT7 inhibitor 97491	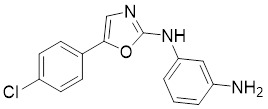	Other	SIRT7	Uterine cancer	325 nM	292

**Table 7:** Combination Therapeutics with SIRT modulators

SIRTi	Drug	Dosage	Cancer type	Therapeutic mechanism	Refs
EX-527	Sorafenib	Sfb 2 μM + EX 40 μM	HCC	EX-527 enhances apoptotic effects of sorafenib	312
Cambinol	Sorafenib	Sfb 2 μM + Camb 50 μM	HCC	Cambinol enhances apoptotic effects of sorafenib	312
Cambinol	Paclitaxel	PAX: Camb = 1:1	TNBC	Increases in pharmacological effects	309
EX-527	Cisplatin	Unknown	NSCLC	Exerts synergistic anti-tumor effects	309
EX-527	Erlotinib	Unknown	NSCLC	Exerts synergistic anti-tumor effects	309
SRT501	Pabinostat	Unknown	Unknown	Enhances synergistic anticancer effects	137
AGK2	DCA	AGK2: DCA = 1:1000	NSCLC	Leads to synergistic killing of NSCLC cells	311
Sirtinol	DCA	Sirtinol: DCA = 1: 1000	NSCLC	Induces G1 phase block	311
amurensin G	Doxorubicin	Unknown	Breast cancer	Downgrads multidrug resistance	306

## Abbreviations

1,4-DHP: 1,4-dihydropyridine
5-FU: 5-fluorouracil
ACAT1: acetyl-CoA acetyltransferase 1
ACC1: acetyl coenzyme A carboxylase
ADC: antibody-drug couplings
ADM: adenohypophysis to ductal chemotaxis
AMC: aminomethylcoumarin
AML: acute myeloid leukemia
APE1: acid endonuclease-1
ARF: alternative reading frame
Bax: BCL2-associated X protein
CBL: Casitas B-lineage lymphoma
CDK2: cycle protein-dependent kinase 2
circRNAs: circular RNAs
CLL: lymphocytic leukemia
CML: chronic myeloid leukemia
CRC: colorectal cancer
CtBP: C-terminal binding protein
CYPD: Cyclophilin D
DBC1: breast cancer deletion 1
DDX3X: DEAD-box helicase 3
DLBCLs: Diffuse large B cell lymphomas
DPT: deoxypodophyllotoxin
DSB: double-strand break
EGF: epidermal growth factor
EMT: epithelial-mesenchymal transition
FASN: fatty acid synthase
FGL1: fibrinogen-like protein 1
FOXO3a: Forkhead box protein O3
GBM: glioblastoma multiforme
GDH: glutamate dehydrogenase
GLDC: glycine decarboxylase
GLS: glutaminase
GLUD1: glutamate dehydrogenase 1
GLUT1: glucose transporter-1
GNPAT: glycerol O-acyltransferase
GP: grape powder
GPX4: glutathione peroxidase 4
GSK-3β: glycogen synthase kinase-3β
H3K9me1: H3K9 monomethylation
HAT: histone acetyltransferases
HCC: hepatocellular carcinoma
HDACs: histone deacetylases
HIC1: hypermethylation in cancer 1
HIF1α: Hypoxia-inducible factor 1α
HINT1: histidine triad nucleotide-binding protein 1
HPLC: high performance liquid chromatography
HR: homologous recombination
HRE: hypoxia response element
HTS: high-throughput screening
HUR: the tumor suppressor
IBC: Isobavachalcone
IDH1: isocitrate dehydrogenase 1
IDH2: isocitrate dehydrogenase 2
IGF-I: insulin-like growth factor-I
INZ: Inauhzin
KIF23: kinesin family member 23
KMP: Kaempferol
LAP2α: lamina-associated polypeptide 2 α
LDH: lactate dehydrogenase
LDHB: lactate dehydrogenase B
LKB1: Liver kinase B1
lncRNA: long-stranded noncoding RNA
LONP1: Lon protease-1
MAPK: mitogen-activated protein kinases
MCU: mitochondrial calcium uniporter
MDM2: Murine double minute 2
miRNAs: microRNAs
MM: multiple myeloma
MnSOD: mitochondrial superoxide dismutase 2
MTHFD2: methylenetetrahydrofolate cyclic hydrolase 2
mTORC1: mammalian target of rapamycin protein complex 1
Mxd1: MXD dimerization protein 1
NAD+: nicotinic adenine dinucleotide+
NAM: Nicotinamide
NAT10: N-acetyltransferase 10
NDRG1: N-myc downstream regulated gene 1
NER: nucleotide excision repair
NHEJ: non-homologous end-joining
NLS: nuclear localization signal
NOX2: NADPH oxidase 2
NSCLC: non-small cell lung cancer
OC: ovarian cancer
OCR: oxygen consumption rate
OS: ovarian cancer
OSCC: oral squamous cell carcinoma
OXPHOS: oxidative phosphorylation
PAX: Paclitaxel
PCAF: P300/CBP-associated factor
PCDH20: procalcitonin 20
PD: Parkinson's disease
PD: pharmacodynamic
PDAC: pancreatic ductal adenocarcinoma
PDC: pyruvate dehydrogenase complex
PDHA1: pyruvate dehydrogenase α1
PDK1: pyruvate dehydrogenase kinase-1
PD-L1: programmed death ligand 1
p-ERK: phosphorylation of ERK
PFK1: phosphofructokinase-1
PGAM: Phosphoglycerate amphoteric acid mutase
PHD: prolyl hydroxylase
PK: pharmacokinetic
PKM2: pyruvate kinase M2
Prdx-1: protein peroxiredoxin1
PTEN: phosphatase and tensin homolog
PTM: post-translational modification
PYCR1: pyrroline-5-carboxylic acid reductase 1
R5P: ribulose-5-phosphate
RIF1: replication timing regulator 1
SAMHD1: Sterile alpha motif and HD domain-containing protein 1
SHMT2: hydroxymethyltransferase 2
Sir2: silent information regulator 2;SIRTs: sirtuins
Skp2: S-phase kinase-associated protein 2
SLC25A51: solute carrier family 25 member 20
SOD2: superoxide dismutase 2
T-ALL: T-cell acute lymphoblastic leukemia
TGF: transforming growth factor
TKT: transketolase
TM: Thiomyristoyl
TNBC: triple-negative breast cancer
WRN: Werner helicase
XRCC1: X-ray cross-complementarity-1
